# The Case for Pneumococcal Surface Protein A (PspA): A Comprehensive Review of a Leading Candidate in Pneumococcal Vaccine Research

**DOI:** 10.3390/vaccines14050374

**Published:** 2026-04-23

**Authors:** Bárbara Milani, Nauany Reis Zordan, Rodrigo Hipolito Penha, Thaisy Pacheco, Lucio Fábio Caldas Ferraz, Thaís Manzano Parisotto, Thiago Rojas Converso, Michelle Darrieux

**Affiliations:** Laboratório de Microbiologia Molecular e Clínica, Universidade São Francisco, Bragança Paulista 12916-900, São Paulo, Brazil; barbara.froes@usf.edu.br (B.M.); thais.parisotto@usf.edu.br (T.M.P.); thiago.converso@usf.edu.br (T.R.C.)

**Keywords:** *Streptococcus pneumoniae*, pneumococcal vaccines, pneumococcal surface protein A (PspA), animal model

## Abstract

*Streptococcus pneumoniae* remains a leading cause of morbidity and mortality worldwide, with current polysaccharide-based vaccines offering limited serotype coverage, high production costs, and reduced efficacy in vulnerable populations. These limitations have prompted the search for conserved pneumococcal proteins as universal vaccine candidates. Among them, pneumococcal surface protein A (PspA) stands out as a major virulence factor, present in virtually all clinically relevant strains, and capable of interfering with complement activation, opsonophagocytosis, and host defense mechanisms. Over three decades of research have demonstrated PspA’s strong immunogenicity, protective efficacy in multiple animal models, and safety in early-phase clinical trials. Here, we critically review advances in PspA-based vaccine development, including recombinant protein fragments, fusion constructs, nanoparticle formulations, and live-vector platforms. We highlight the structural and immunological determinants underlying its protective potential, while discussing major challenges such as antigenic variability and cross-reactivity across pneumococcal strains expressing distinct PspA clades. By integrating recent experimental and translational findings, this review outlines the opportunities and obstacles for the implementation of serotype-independent PspA-based vaccines.

## 1. Introduction

*Streptococcus pneumoniae* (pneumococcus) is an extracellular pathogen that primarily colonizes nasopharynx, from where it can spread to other sites, causing local and/or systemic infections such as pneumonia, bronchitis, brain abscess, otitis media, septicemia, and meningitis. Globally, *S. pneumoniae* is the leading cause of morbidity and mortality among lower respiratory tract infections (LRTIs); it is responsible for approximately 9 million infections each year, with over 1.1 million related deaths, primarily affecting children, immunocompromised individuals, and the elderly [1,2]. Due to its high global mortality rate, it has been classified as a medium-priority pathogen by the World Health Organization (WHO) for research and development of new antibiotics [3]. The increasing prevalence of antibiotic-resistant strains exacerbates the situation, limiting therapeutic options. Currently, approximately 30% of severe infections caused by pneumococci show resistance to one or more antibiotics [4,5]. Prevention of pneumococcal infections is currently achieved through immunization using capsular polysaccharides from *S. pneumoniae*, alone or conjugated with carrier proteins [6]; however, the high variability of the capsule, combined with technical challenges in vaccine production, such as high costs and difficulties in implementation in developing countries, limits vaccine effectiveness and coverage [7,8]. Therefore, serotype-independent, protein-based formulations have been investigated against *S. pneumoniae* [9].

Pneumococcal surface protein A (PspA) is an important virulence factor in *S. pneumoniae.* It is the most prevalent member of the choline-binding protein family (CBPs), which includes surface molecules attached to the phosphorylcholine moieties of teichoic acids in the cell wall [10,11]. The immunogenic potential of PspA was identified over three decades ago, with the demonstration that two monoclonal antibodies against PspA protected mice from fatal infection with three different pneumococcal strains expressing two capsular serotypes [10,12].

**Figure 1:**
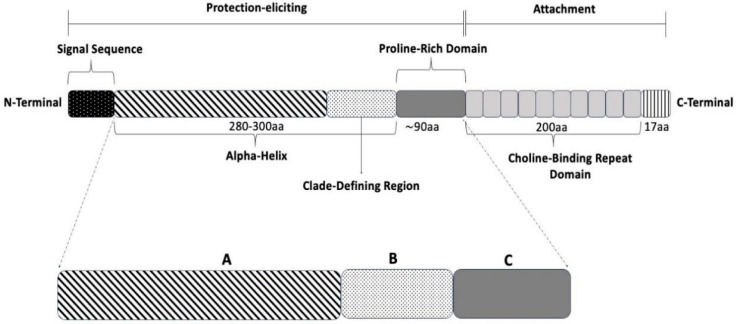
Schematic representation of the PspA structure. The N-terminal signal sequence, the α-helical region with 280–300 aa (A) including a clade-defining region (B), the proline-rich domain of approximately 90 amino acids (C), the choline-binding repeat domain with about 200 aa, and a short C-terminal tail of 17 aa are shown.

Structurally, PspA contains three distinct regions (Figure 1): an N-terminal alpha-helical domain, a proline-rich domain, and a choline-binding domain at the C-terminus. The first domain is the exposed part of the molecule, and the most variable portion of the molecule. It includes a clade-defining region (CDR) used to classify PspA into 3 families and 6 clades. Family 1 includes clades 1 and 2; family 2 includes clades 3, 4, and 5; and family 3 includes clade 6 [13]. Primary amino acid sequences within the same clade show ≥90% identity in the CDR, while between different families, this identity is ≤55% [13,14,15]. Most pneumococcal strains express PspA from families 1 or 2. The level of cross-reactivity varies among different PspAs, with higher reactivity within proteins of the same family. The proline-rich region (PRR, or region C) is located between the N-terminal helical segment and the C-terminal domain, and is characterized by short repeats, in which proline residues occur every three or four amino acids. In approximately half of the described PspAs, these repeats are interrupted by a highly conserved sequence of amino acids lacking proline, known as the non-proline block (NPB), which is considered an important antigenic fragment [16]. The C-terminal region of PspA is responsible for the protein interaction with choline residues in the bacterial membrane, forming a conserved structural domain present in all variants of this protein, followed by a short hydrophobic tail.

PspA contributes to pneumococcal virulence through multiple mechanisms. It protects the bacterium from phagocytosis and interferes with complement system activation, reducing opsonophagocytosis [17]. PspA also acts as an adhesin by binding to host proteins such as glyceraldehyde-3-phosphate dehydrogenase on dying lung cells and lactate dehydrogenase (LDH), which supports bacterial growth by enhancing energy production during infection [18]. Another important trait in PspA relies on its ability to interact with human lactoferrin, a component of the innate mucosal defense system. This interaction reduces the bactericidal efficacy of apo-lactoferrin (Apo-hLf)—the iron-free form of the molecule—promoting pneumococcal survival. It has been shown that the presence of PspA on the bacterial surface inhibits the lytic activity of lactoferrin, protecting the pathogen, whereas anti-PspA antibodies enhance the bactericidal effect of lactoferrin against *S. pneumoniae* [19,20,21]. Recent studies have also demonstrated that PspA can protect *S. pneumoniae* against the microbicidal effects of indolicidin, a cationic antimicrobial peptide belonging to the cathelicidin family. Mass spectrometry analyses have demonstrated a direct interaction between PspA and indolicidin, supporting the hypothesis that PspA functions as a molecular net. By sequestering cationic peptides, it effectively prevents their access to the bacterial membrane, thereby mitigating membrane disruption and preserving cellular integrity [22].

Due to its relevant role in pneumococcal virulence, PspA has been investigated in numerous studies as the leading candidate in serotype-independent vaccines, particularly using its N-terminal region, which contains most of the protective epitopes [16]. Immunization of mice elicited both mucosal and systemic immune responses, providing protection against nasopharyngeal colonization, pulmonary infection, and sepsis following challenge with *S. pneumoniae* [23]. In chinchillas, PspA has also demonstrated protection against otitis media [24]. Recently, the vaccine potential of PspA was evaluated in a nasal vaccine study model using non-human primates, with encouraging results [25]. Beyond its well-documented protective efficacy in animal models, PspA has advanced to phase I clinical trials [26,27,28], underscoring its promise as a vaccine candidate against *S. pneumoniae*.

Given the considerable vaccine potential demonstrated by PspA, this review aims to critically examine the immune responses elicited by this surface protein in various formats—including as a standalone antigen, in fusion constructs, or in combination with other pneumococcal components. Furthermore, the review seeks to analyze the quality and breadth of the immune responses generated, while also addressing the current limitations and challenges associated with the use of PspA as a vaccine candidate. The following topics describe different infection models using PspA as a vaccine candidate, which represent the main diseases caused by this pathogen. They have also been summarized in Table 1, Table 2, Table 3 and Table 4.

## 2. Sepsis

Various vaccine formulations based on PspA have been investigated as strategies to prevent systemic infections caused by *S. pneumoniae*, particularly in murine models of sepsis. These formulations include isolated protein fragments, combinations or fusions with other antigens, as well as the use of distinct vaccine vectors and adjuvants [30,40,47,53,54,55,77]. Table 1 summarizes the studies evaluating the protective role of PspA against sepsis.

### 2.1. Full Length PspA and PspA Fragments

Initial investigations of PspA as a vaccine candidate against systemic infection employed the full-length protein isolated from non-encapsulated *S. pneumoniae* strains. This approach proved effective in murine sepsis models, conferring protection even against high challenge doses [78,79]. However, technical limitations associated with native protein purification—such as its aggregation propensity—prompted the exploration of novel vaccine formulations [79,80]. Subsequent studies demonstrated that fragments derived from the N-terminal region of PspA, particularly those comprising the first 245 amino acids, retained their protective immunogenicity [79,81,82]. The foundational study from McDaniels et al. identified specific PspA regions that are recognized by monoclonal antibodies (MAbs) produced in mice immunized with purified PspA [83]. The epitopes were located on the alpha-helical N-terminal region of the molecule, more specifically in aa 1–115 and 192–260. Five out of nine MAbs were protective against lethal pneumococcal challenge, with four of those binding to aa 192–260. Furthermore, mice immunized with a recombinant fragment encompassing this region survived challenge with a serotype 2 highly virulent pneumococcal isolate. Interestingly, this protective region of PspA was located adjacent to the bacterial cell wall and not as exposed as more N-terminal domains.

The encouraging results prompted a series of further studies evaluating the immunogenicity of PspA fragments within the N-terminal region of the molecule [15,84,85,86]. Two consecutive works have demonstrated that mice immunized with truncated PspAs are protected against lethal challenge with pneumococci of different serotypes. Tart et al. [84] identified residues 192–588 as the optimal region for protection, while Roche et al. [15] refined this by identifying that the C-terminal end of the alpha-helical domain (the clade-defining region) and the proline-rich region (PRR) are the most potent for broad protection. These studies were complemented with works investigating the cross-reactivity among PspAs from different clades and families, which showed variable levels of cross recognition of antibodies against PspA. Although this variation roughly followed the sequence similarities of PspA molecules, later work identified variable levels of cross-reactivity within each family, and identified two N-terminal fragments from family 1 [14] and two fragments from family 2 [35] that induced antibodies with higher levels of cross-recognition of clinical isolates. Finally, overlapping peptide screening techniques confirmed that fragments of 100 amino acids within the N-terminal region can induce functional antibodies against both linear and conformational epitopes, and the latter are required for protection against pneumococcal challenge, making them preferred targets for multi-epitope formulations [87,88].

While the vast majority of PspA formulations include the N-terminal region of the protein, few studies have focused on the proline-rich region of PspA (named PRR) as a vaccine candidate against systemic *S. pneumoniae* infections, given its conserved and immunogenic epitopes [16,29,30]. This approach was first evaluated by Girgis et al. as an isolated antigen, with BALB/c mice receiving three subcutaneous doses of recombinant PRR protein adsorbed into aluminum hydroxide (Al(OH)_3_) followed by intraperitoneal challenge with a clinical serotype 19F strain. Passive immunization with anti-PRR antibodies—despite generating high antibody titers—failed to provide significant protection (0% survival in all vaccinated groups). This outcome was attributed to the strain’s thick polysaccharide capsule potentially masking PspA epitopes and impairing humoral response efficacy [30]. Building on these findings, Tamborrini et al. investigated synthetic virus-like particles (SVLPs) containing proline-rich region epitopes in a murine systemic sepsis model using *S. pneumoniae* serotype 1 (strain 1577). NMRI mice were immunized with SVLPs containing either: (i) CCL-PR1 (N-terminal proline-rich region epitopes), CCL-NPB (conserved non-proline block), or their combination; or (ii) heterologous epitopes CCL-PR2/N (N-terminal region variant) or CCL-PR2/C (C-terminal variant), where CCL refers to the Coiled-Coil-Like nanoparticle platform. Following two immunizations, intravenous challenge with 10^4^ CFU of strain 1577 revealed significant morbidity delay in CCL-PR1, CCL-NPB, and CCL-PR1+CCL-NPB, but not in recombinant rPspA controls. Among heterologous epitopes, only CCL-PR2/C significantly improved survival, suggesting protective potential for specific fragments like NPB and C-terminal variants [29].

The investigation by Daniels et al. examined the immunogenicity and protective efficacy of distinct fragments from the proline-rich region in CBA/N mice using a systemic pneumococcal infection model. Three recombinant fragments were evaluated: PR+NPB (containing both the proline-rich region and the Non-Proline Block subregion), PR-NPB (comprising only the proline-rich region without NPB), and NPB alone (the isolated Non-Proline Block). Mice received three intraperitoneal doses of 10 µg protein adsorbed to Al(OH)_3_, followed by lethal challenge with heterologous encapsulated strains of serotype 3 (WU2 and 3JYP2670) and serotype 6A (BG12730), which express different PspA variants. The PR+NPB fragment induced significant protection (70–80% survival across all three strains), whereas PR-NPB and NPB alone failed to confer effective protection. These results establish that the NPB subregion, when maintained in its native context within the proline-rich region, is essential for eliciting functional protective immunity, identifying it as a conserved epitope of interest for C-terminal-based PspA vaccine formulations [16].

In conclusion, the proline-rich region of PspA is an interesting strategy to prevent pneumococcal sepsis, especially when the NPB was included in the formulation, but protection is dependent on the bacterial strain.

### 2.2. Multicomponent and Chimeric Vaccines

PspA has been extensively investigated in multicomponent formulations, in combination or in fusion with different pneumococcal antigens. Formulations combining PspA with pneumococcal histidine proteins PhtB and PhtE, PspC and PdB (a detoxified form of pneumolysin) were tested in a murine model of invasive infection. Mice were immunized with three doses of proteins in different combinations, using Alum as adjuvant, and challenged with virulent type 2 and 6A strains. Overall, the combinations were more protective than each protein alone, with the combination of PdB, PspA, and PspC leading to the highest protection [89].

Another approach employed native proteins purified from *Streptococcus pneumoniae* using different methods. To evaluate the protection conferred by a combination of pneumococcal choline-binding proteins—CBPs, including PspA, PspC, LytA, B and C, as well as other less prevalent proteins—the study by Dion & Ashurst investigated the immunization of BALB/c mice with native proteins extracted from different *S. pneumoniae* strains, including non-encapsulated mutant strains that expressed different PspAs as well as PspA-negative strains. The animals were immunized subcutaneously, without the use of adjuvants, with three doses containing 1 µg of CBPs eluted with 2% choline chloride from strains cultured in THY medium. Three weeks after the last immunization, mice were challenged by intranasal aspiration with strains A66.1 (serotype 3, PspA clade 2) or ATCC 6303 (serotype 3, PspA clade 5). Against the A66.1 challenge, survival rates were 100% for Rx1 CBPs, 92% for Rx1PspA4, 83% for Rx1ΔPspA, 75% for Rx1PspA1, and 42% for rPspA4, while the control group (saline) showed only 8% survival. In the ATCC 6303 challenge, the highest survival rates were observed in groups immunized with Rx1PspA4 (58%) and Rx1ΔPspA (42%) CBPs, followed by Rx1PspA1 (33%), rPspA4 (17%), Rx1 (8%), and saline (0%). These data indicate that immunization with native CBPs, even in the absence of adjuvant, can confer significant protection against pneumococcal sepsis, with variable efficacy depending on the antigenic composition of proteins extracted from each strain [42]. Furthermore, the higher survival rates found in groups immunized with CBPs including PspA reinforces its contribution to protective immune responses.

A second study evaluating combinations of native proteins used *S. pneumoniae* TIGR4 lysates enriched for surface proteins by a chromatography step after culture under conditions that induce expression of heat shock proteins as immune adjuvants [38]. The resulting multiantigen vaccine (MAV) was enriched for PspA and pneumolysin, among other proteins. Immunization with MAV induced robust antibody responses to pneumococci of different serotypes, which passively protected rodents against lethal infection with diverse pneumococci.

One of the main limitations of PspA as a vaccine candidate is its high structural variability, which can compromise the breadth of protection when only a single fragment or variant of the protein is used. As a strategy to overcome this barrier, different groups have investigated the development of PspA fusion proteins, in which immunogenic fragments are combined to broaden cross-reactivity. In the study by Darrieux et al. formulations containing the N-terminal region of PspA from different clades were constructed, resulting in the antigens PspA1ABC (clade 1), PspA3ABC (clade 3), as well as the hybrids PspA1ABC-4B (clade 1 fused to fragment B of clade 4) and PspA1ABC-3AB (clade 1 fused to fragments A and B of clade 3). BALB/c mice immunized subcutaneously with these formulations and challenged with representative strains of clades 1, 2, 3, and 4 showed protection directly related to the similarity between the vaccine fragment and the clade expressed by the bacterium, with particular emphasis on the hybrids, which conferred broader protection and correlated with increased C3 deposition on the bacterial surface [31]. Similarly, Piao et al. [33] evaluated PspA fusion proteins constructed from fragments of the N-terminal region (α-helical domain and proline-rich region) of different clades from families 1 and 2. The experimental design was comparable, differing only in the vaccination scheme (the combined use of CpG ODN and Al(OH)_3_ as dual adjuvants in C57BL/6J mice) and in the intranasal challenge with strains expressing PspA from clades 1 to 5. Results showed that, as observed by Darrieux et al., protection depended on the choice of fragments included in the chimeric protein: the PspA3+2 formulation conferred protection against all five clades tested, whereas PspA2+4 and PspA2+5 failed against strains from clades 1 and 3. These findings indicate that the inclusion of fragments from clades 3 and 2 in the PspA3+2 construct resulted in a broader range of cross-reactivity and protection in the murine pneumococcal sepsis model [33].

The efficacy of multivalent formulations combining PspA fragments with CbpA (currently designated PspC) and a mutated pneumolysin toxin (L460D) was evaluated in murine sepsis models. Among the PspA fragments tested, two were particularly noteworthy: H70 (containing the SM1 peptide and proline-rich region) and CD2 (comprising only the proline-rich region). These fragments were fused with either the YLN fusion protein (L460D linked to two CbpA domains, YPT and NEEK) or L460D alone. CD1 mice immunized intraperitoneally with these formulations were challenged with strains D39 (serotype 2), P9 (serotype 6A), or 1861 (serotype 1). The trivalent H70+YLN formulation provided significant protection against all tested strains, particularly against P9—reducing meningitis incidence from 50% in control mice to 20% in immunized animals. In intravenous sepsis models using strains DBL6A (serotype 6B) and A66.1 (serotype 3), H70+YLN again demonstrated superior protection compared to individual antigen components [34]. Collectively, these studies highlight the proline-rich region’s potential, particularly through conserved NPB fragments and multivalent formulations, for inducing protective immunity in pneumococcal sepsis models. The strategic combination of this region with other immunogenic proteins and adjuvants appears crucial for achieving broad efficacy against diverse *S. pneumoniae* serotypes [34].

Studies analyzing a chimeric formulation combining the N-terminal region of PspA with detoxified pneumolysin, demonstrated high efficacy of this formulation, with detoxification eliminating the cytolytic activity of the toxin while preserving its immunogenicity [32,40]. In the study by Goulart et al. [32] PspA was fused to PdT, a non-toxic pneumolysin derivative generated by site-directed mutagenesis of the ply gene (L460D). BALB/c mice immunized subcutaneously with three doses and challenged intravenously with serotype 3 strains showed that PdT alone did not confer protection. In contrast, the chimeric formulations PspA1-PlD1 and PspA1-PlD2 elicited a strong antibody response with enhanced complement deposition and opsonophagocytosis, leading to 100% survival, whereas PspA2-PdT induced partial but significant protection compared to PdT alone. In contrast, Milani et al. [40] demonstrated that the fusion of the N-terminal region of PspA1 to the detoxified pneumolysin PlD1 (H367R mutant) resulted in 90% survival against heterologous serotype 3 challenge (3JYP2670), surpassing both PspA1 alone and co-administration with PlD1 (50% survival). This formulation also induced efficient C3 deposition and produced cross-reactive antibodies (32). Collectively, these findings highlight that detoxified pneumolysin significantly enhances the protective efficacy of PspA-based vaccines in systemic infection models These findings indicate that detoxified pneumolysin plays a relevant role when genetically combined with PspA, enhancing the efficacy of the immune response in sepsis models [32,40].

Similarly, Zane et al. evaluated the impact of including either flexible or rigid linkers between the PspA and PdT proteins. The authors investigated how inserting peptide linkers—a rigid one (RL) or a flexible one (FL)—between two fused pneumococcal proteins (PspA and PdT), using the N-terminal region of PspA, could influence the structural stability and production of the candidate vaccine. Using both in silico and experimental approaches, the researchers compared three versions of the protein: without a linker, with a rigid linker, and with a flexible linker, and assessed their performance in production models and protective efficacy in specific pathogen-free (SPF) female BALB/c mice. The animals were immunized subcutaneously in a three-dose regimen, spaced 15 days apart, with either rPspA-FL-PdT or rPspA-RL-PdT, using sterile 0.9% saline and Al(OH)_3_ as an adjuvant. The adjuvant alone in saline served as a negative control. Twenty-one days after the third dose, the animals were challenged intranasally with *S. pneumoniae* strain A66.1. Specific IgG antibody levels were measured 15 days after each immunization. The results showed that, for anti-PspA IgG, antibody levels against all fusion proteins were similar, with more noticeable differences between the first and second doses, reaching a plateau after the second dose. The third dose increased anti-PspA antibody levels only in the groups immunized with rPspA-FL-PdT. For anti-PdT IgG, marked differences were observed, with higher levels after the third dose. Immunization with rPspA-PdT without a linker showed stability issues that compromise large-scale production, making the inclusion of linkers a promising strategy to overcome this limitation. Both versions provided 100% protection against a lethal intranasal challenge with *S. pneumoniae*, showing that linker inclusion—especially the flexible type—enhances pharmacological traceability without impairing immunological efficacy, representing a step forward in producing safe and stable fusion vaccines [41]. Thus, the production of rPspA-FL-PdT enables the delivery of two antigens in a single process, which is advantageous from both economic and bioprocessing standpoints, while also yielding a more stable molecule compared to the linker-free version [40,41].

Converso et al. [90] developed a recombinant vaccine based on a chimeric protein containing the N-terminal region plus the proline-rich region of PspA (St P490 strain, family 2) in fusion with PotD (St 540/99 strain)—a conserved pneumococcal polyamine-binding lipoprotein involved in polyamine transport and associated with bacterial virulence [91]. The resulting formulation, rPspA-PotD, was tested in a murine model of sepsis following intranasal challenge with two virulent *S. pneumoniae* strains, ATCC6303 (serotype 3, PspA clade 5) and A66.1 (serotype 3, PspA clade 2). The rPspA-PotD vaccine conferred significant protection, with a survival rate of 90% against ATCC6303 and 90% against A66.1, demonstrating similar efficacy to isolated PspA protein, and superior to isolated PotD, which conferred no protection. Furthermore, the hybrid formulation induced high levels of specific IgG antibodies, induced antibodies that recognized different PspA variants across various pneumococcal strains and promoted increased in vitro phagocytosis. A reduction in nasopharyngeal colonization after challenge was also observed. These data confirm the ability of the rPspA-PotD chimeric protein to induce a comprehensive protective immune response, both against lethal systemic infection and mucosal colonization [35].

The pneumococcal adhesin PsaA—a surface lipoprotein involved in manganese transport and adhesion to host cells [92]—has also been used in fusion with PspA. Yu et al. tested a bivalent formulation composed of the PsaA-PspA23 fusion protein (with epitopes from clades 2 and 3) co-administered with the PspA4 protein (clade 4), both adsorbed into Al(OH)_3_. Female BALB/c mice were immunized subcutaneously with three doses of the vaccine formulation at two-week intervals. Two weeks after the last immunization, the animals were challenged intranasally with virulent *S. pneumoniae* strains representing PspA clades 1 to 5, including strains ATCC 6312 (clade 1), ATCC 6304 (clade 1), ATCC 10813 (clade 2), ATCC BAA-334 (clade 3), and ATCC 6303 (clade 5). The group vaccinated with the PsaA-PspA23+PspA4 formulation showed over 50% survival in all challenges, with significantly greater protection compared to control groups (PBS and PPV23), regardless of the challenging strain’s clade of origin [36]. These results demonstrate that co-administration of PspA antigens from different families, along with PsaA, can provide robust cross-protection against lethal systemic pneumococcal infection.

In an innovative approach, Suzuki et al. developed a fusion vaccine combining PspA with the receptor-binding domain (RBD) of parvovirus B19. The RBD corresponds to the VP1 unique region (VP1u) of the viral capsid, which contains the main neutralizing epitopes and is essential for viral attachment and entry into host cells, making it a strategic target for vaccine development. The parvovirus B19 is a single-stranded DNA human pathogen that can cause different clinical manifestations, ranging from infectious erythema in children to severe complications. Considering that both B19 and *S. pneumoniae* are relevant respiratory mucosal pathogens capable of causing serious diseases, the fusion of the B19 RBD with pneumococcal PspA was proposed as an innovative approach to induce protective immunity against two distinct agents in a single formulation. The chimeric protein RBD-PspA was expressed in *E. coli*, purified, and used for intramuscular immunization of BALB/c mice in three doses administered at two-week intervals. The aim was to evaluate whether the formulation could induce protective immunity against two distinct pathogens. To assess protection against pneumococcal pneumonia, two weeks after the final dose animals were inoculated intranasally with *S. pneumoniae* serotype 3 (strain ATCC6303) and monitored for 14 days. Mice immunized with RBD-PspA showed 100% survival, similarly to the group immunized with PspA alone, whereas all unvaccinated control animals succumbed to infection. Moreover, the RBD-PspA formulation induced high serum IgG levels against both RBD and PspA, accompanied by class switching from IgM to IgG, an effect not observed with RBD alone. In vitro assays using splenocytes indicated that immunization with the chimeric protein activated PspA-specific helper T cells, which secreted IL-5 and facilitated class switching also against RBD, thereby conferring dual immunity to both antigens [37].

Finally, in silico analysis of prevalent epitopes in PspA has led to the design of chimeric molecules with increased protective efficacy. Afshari et al. employed a bivalent vaccine formulation containing the chimeric protein PspA1-5c+p—which incorporates epitopes from regions B and C of the five major PspA clades—combined with the highly conserved PhtD protein. BALB/c mice immunized intraperitoneally with this formulation exhibited 100% survival following intraperitoneal challenge with strain ATCC 49619 (serotype 19F), along with complete bacterial clearance in the spleen and significant reduction in blood bacterial load. When compared to formulations containing either antigen alone, the PspA+PhtD combination showed superior efficacy, clearly demonstrating the advantage of this multi-epitope approach [39].

### 2.3. Vectored Vaccines and Nanoparticles

Recombinant bacteria such as BCG, *Salmonella* and *Lactobacillus* (the latter two are discussed in the “mucosal vaccines topic”) have been used as live vectors to deliver PspA. Goulart et al. investigated the use of BCG as a live vaccine vector expressing pneumococcal proteins for protection against lethal *S. pneumoniae* infection. C57BL/6 mice were immunized with recombinant BCG (rBCG) strains individually expressing SP0148, SP2108, or the PspA-PdT fusion protein, or with a mixture of these strains (rBCG Mix), followed by a booster dose containing the respective recombinant proteins (rMix). The SP0148 and SP2108 proteins, individually expressed by recombinant *Mycobacterium bovis* (Bacillus Calmette–Guérin, BCG) strains, are not variants of PspA but rather distinct pneumococcal surface proteins associated with transport and adhesion mechanisms, respectively. Only the rBCG PspA-PdT construct expresses pneumococcal surface protein A (PspA) fused to detoxified pneumolysin (PdT). The sepsis model was induced by pulmonary aspiration of the virulent WU2 strain (serotype 3), and animals were monitored for 15 days to assess survival rate. Results showed that only co-administration of multiple pneumococcal antigens expressed in a live vector followed by a booster with recombinant proteins (rBCG Mix/rMix), was able to significantly protect mice against lethal challenge, with 90% survival, compared to total mortality observed in the control group [43]. These findings underscore the potential of the multivalent approach based on recombinant proteins delivered via BCG vector as a promising serotype-independent vaccine candidate for the prevention of pneumococcal sepsis.

Since BCG is a vaccine approved for use in newborns in many countries, recombinant *M. bovis* expressing the fusion protein between PspA-PdT has been evaluated in a neonate mouse model. The mice received a single intraperitoneal dose of rBCG-PspA-PdT on the fifth day of life, followed by a booster with the aluminum-adjuvanted recombinant protein on the 12th day. Intranasal challenge with the WU2 strain (serotype 3; clade 2) at 21 days resulted in 100% survival, demonstrating the formulation’s ability to induce functional protective immunity at a critical phase of immune development [47].

Other studies have investigated the use of genetically inactivated bacteria as vaccine delivery systems. Castro et al. explored the potential of recombinant *Bordetella pertussis* expressing PspA4Pro as a delivery system/adjuvant for immunization against *S. pneumoniae*. For this purpose, *B. pertussis* strains producing PspA from clade 4 (PspA4Pro), fused to the N-terminal region of filamentous hemagglutinin (Fha44), were used to generate the wP^PspA4Pro formulation. Female BALB/c SPF mice were immunized subcutaneously under different regimens: wP^PspA4Pro alone; purified PspA4Pro protein; or the fusion protein Fha44:PspA4Pro. In the prime–boost strategy, animals received the recombinant wP^PspA4Pro followed by booster doses with purified PspA4Pro or saline. The Fha44:PspA4Pro group received two subcutaneous doses of the fusion protein, 15 days apart. Isolated PspA4Pro and Fha44 were used as controls. Twenty-one days after the final immunization, mice were challenged intranasally with *S. pneumoniae* ATCC6303 (serotype 3). The results showed that immunization with wP^PspA4Pro induced low anti-PspA4 IgG levels and did not protect against the lethal pneumococcal challenge. Purified PspA4Pro induced higher antibody levels and greater protection against pneumococcal infection than the prime–boost strategies, with sera from PspA4Pro-immunized mice being the only ones to promote significant C3 complement deposition on the pneumococcal surface. Finally, purified Fha44:PspA4Pro induced high anti-PspA4Pro IgG titers but failed to confer protection, suggesting that antibodies elicited by the fusion protein were not directed against protective epitopes [44].

Figueiredo et al. [45] evaluated the efficacy of chitosan-incorporated nanoparticles (NPs) as adjuvants in different PspA-based formulations: (1) PGA-co-PDL/HCl-CS polymeric NPs with surface-adsorbed PspA, and (2) PLGA/HCl-CS NPs encapsulating PspA. Prior to vaccination, mice were anesthetized intraperitoneally with a xylazine/ketamine solution (20 mg/kg xylazine and 100 mg/kg ketamine). Female BALB/c mice received pulmonary mucosal vaccination with either empty NPs or NPs containing 2 μg or 6 μg PspA4Pro. Control groups included subcutaneous (sc) injection of saline or purified PspA4Pro (2 μg or 6 μg in 100 μL), plus pulmonary instillation of purified PspA4Pro (2 μg or 6 μg in 50 μL, administered to one nostril under anesthesia). Mice were immunized twice at 14-day intervals and challenged 21 days post-final immunization. Results demonstrated that both formulations induced PspA-specific serum IgG production but with distinct functional profiles. Mice immunized with encapsulated PspA (PLGA/HCl-CS) showed 100% survival after lethal *S. pneumoniae* challenge, versus 83% survival with adsorbed PspA (PGA-co-PDL/HCl-CS). The encapsulated formulation more efficiently activated dendritic cells, with elevated expression of activation markers (CD40 and MHC class II), suggesting enhanced antigen presentation. Structural analysis revealed that encapsulated PspA maintained structural integrity post-release, while surface adsorption impaired functional properties like lactoferrin-binding capacity. These findings demonstrate that pulmonary immunization with PspA-containing nanoparticles—particularly encapsulated formulations—provides robust and durable protection against lethal pneumococcal infection [45].

A study by Majumder et al. [46] demonstrated the protective potential of bacterial OMVs carrying a bivalent PspA formulation, including the N-terminal regions of both PspA family 1 and family 2. THs OMVs were produced by an engineered *Yersinia pseudotuberculosis* strain. Intramuscular immunization of mice with OMV-PspA in a prime–boost strategy led to strong antibody production and robust lung-resident memory T cells responses. The immunized mice were protected from both influenza and secondary bacterial infection with heterologous pneumococcal strains. Interestingly, the induction of potent humoral and cellular immune responses in both systemic and respiratory compartments observed in the vaccinated group demonstrates that the intramuscular route is a viable strategy to promote protective immunity in the lungs.

### 2.4. Mucosal Vaccines

Different groups have investigated the potential of PspA-based mucosal vaccines to protect against pneumococcal sepsis. Campos et al. [49] engineered *Lactobacillus casei* to express a recombinant N-terminal fragment of clade 1 PspA. Intranasal immunization with the vaccine (administered on days 0, 1, 14, 15, 28 and 29) induced both mucosal and systemic antibody responses in mice, with increased protection against intraperitoneal challenge with the virulent pneumococcal strain A66.1. The induced antibodies increased complement C3 deposition on the pneumococcal surface, favoring bacterial clearance by phagocytosis.

Continuing the exploration of mucosal live-vectored formulations, a series of studies investigated the immunogenicity and protective efficacy of recombinant attenuated *Salmonella* vaccines expressing PspA [28,48,50,51,52]. Early foundational work by Kang et al. [48] utilized *Salmonella enterica* serovar Typhimurium strains with specific deletions in global regulatory genes, such as Δcya and Δcrp, to ensure attenuation while maintaining immunogenicity. The strains were transformed with plasmids for expression of recombinant PspA N-terminal fragments. Oral delivery of these live vectors successfully induced robust systemic IgG and mucosal IgA responses and protected mice against pneumococcal sepsis. A consecutive study used *Salmonella* strains with regulated delayed attenuation, which provided an optimized release of recombinant PspA, leading to higher IgG levels, increased T cell secretion of IL-4 and IFN-gamma, and enhanced protection against lethal pneumococcal infection [50]. To enhance vaccine coverage, Xin et al. [51] produced recombinant *Salmonella* strains that expressed hybrid molecules combining fragments of family 1 and family 2 PspAs. This multivalent formulation provided broader cross-protection against diverse pneumococcal families. Outer membrane vesicles (OMVs) shed from *Salmonella* have also been used deliver PspA. Intranasal immunization with OMVs harboring PspA conferred significant protection against lethal infection while circumventing the biological safety concerns associated with the shedding of live bacterial vectors [52].

The clinical potential of recombinant *Salmonella* expressing PspA as a vaccine platform was assessed through a Phase I dose-escalation trial by Frey et al. [28]. Using three recombinant attenuated *S. typhi* vectors expressing PspA, the study found the formulations to be generally safe and well-tolerated in healthy adults at doses up to 10^9^ CFU. While the safety of the formulation was confirmed, the results emphasized the need for continued refinement in vector design and antigen presentation to achieve protective threshold levels in clinical settings.

A different strategy used recombinant PspA (clade 2) combined with the pCA adjuvant (Polymeric Caffeic Acid) administered intranasally in a lethal pneumonia/sepsis model using the Xen10 strain (serotype 3). Immunization of BALB/cCrSlc mice with three weekly doses resulted in robust systemic and mucosal immune responses, marked by the presence of IgG and IgA. These IgG antibodies opsonized the bacteria in the bloodstream, allowing for efficient clearance by splenic and hepatic macrophages. As a result, the combined formulation showed a survival rate of 70%, against 10% in the group immunized with PspA alone. These mice also showed a significant delay in the onset of symptoms and a high recovery rate compared to all other groups, reinforcing the potential of the PspA-based nasal vaccine employing this synthetic polymer adjuvant [54].

Of particular interest, the most distinct approach among the models analyzed involved a combination of systemic and mucosal immunization based on PspA variants. BALB/c mice subjected to a prime/boost regimen with subcutaneous application followed by an intranasal booster achieved 100% survival against intranasal challenge with two highly lethal strains—ATCC 10813 (clade 2) and BAA-334 (clade 3). In contrast, exclusively systemic vaccination conferred only 30% protection against the clade 2 strain, while the isolated mucosal route resulted in 80% survival. The combined formulation induced high levels of IgG, IgA, and IL-17A, highlighting the importance of mucosal immunity in protecting against lethal pneumococcal infection [53].

The potential of passive immunization through maternal transfer was investigated using a model where BALB/cByJ female mice were intranasally immunized with rPspA (PspA2/TIGR4) combined with cholera toxin subunit B (CTB). Following intraperitoneal challenge with strain TIGR4, offspring born to and nursed by immunized mothers demonstrated approximately 70% survival and complete absence of bacteremia. The functional immune response involved production of IgG, IFN-γ, and IL-17A, indicating the induction of protective immunological memory [55].

In conclusion, the extensive body of research reviewed here underscores Pneumococcal surface protein A (PspA) as a cornerstone for the development of next-generation, serotype-independent vaccines against invasive pneumococcal disease. Its protective efficacy has been consistently demonstrated across a wide array of formulations, ranging from isolated native proteins to sophisticated recombinant constructs. Data from murine sepsis models highlight that the N-terminal α-helical domain and the proline-rich region (PRR)—specifically the Non-Proline Block (NPB)—are essential for eliciting functional antibodies that promote complement C3 deposition and opsonophagocytosis.

The transition toward multicomponent and chimeric vaccines represents a significant leap in overcoming the structural variability of PspA clades. By fusing PspA fragments with other highly conserved antigens, such as detoxified pneumolysin (PdT), PsaA, or PspC, researchers have achieved synergistic effects that broaden cross-reactivity and significantly enhance survival rates compared to monovalent approaches. Furthermore, the integration of innovative delivery systems—including live bacterial vectors like rBCG and *Salmonella*, as well as nanoparticle encapsulation—has addressed historical challenges regarding protein stability and large-scale bioprocessing.

These diverse strategies have proven effective in both systemic and mucosal immunization routes, providing a dual defense mechanism that reduces nasopharyngeal colonization while clearing lethal bloodstream infections. Collectively, these findings reinforce the critical role of PspA-based formulations as a promising and versatile path toward achieving broad, effective protection against the global burden of systemic *S. pneumoniae* infections.

## 3. Pneumonia

Community-acquired pneumonia remains a significant public health concern, with estimated hospital costs reaching up to 9 billion dollars annually in the United States. The 30-day hospital mortality rate can reach 22%, making it the leading cause of death among infectious diseases. Before the advent of antibiotics, this bacterium was estimated to account for about 95% of cases. Currently, although this proportion has decreased, pneumococcus is still identified in up to 15% of cases in the United States and approximately 27% globally [42]. Vaccines utilizing PspA have emerged as promising alternatives to conjugate vaccines due to their ability to induce cross-protection against different serotypes [93]. Given that *S. pneumoniae* has historically been the bacterial pathogen most frequently associated with pneumonia worldwide, understanding *S. pneumoniae* pulmonary infection models and experimentally evaluating the efficacy of PspA-containing vaccine formulations is crucial for developing more comprehensive immunological strategies. These strategies should provide effective protection against diverse and potentially invasive strains [42]. Table 2 includes a summary of PspA-based vaccines in pneumonia.

The study by Rodrigues et al. [63] developed an experimental pneumococcal pneumonia vaccine based on nanocomposite microparticles (NCMPs) carrying liposomes (LPs) containing two PspA variants: clade 1 (PspA1) and clade 4 (PspA4Pro). Notably, the researchers used recombinant protein fragments corresponding to the mature N-terminal region through the proline-rich domain, encompassing PspA’s major immunogenic epitopes. The liposomes consist of lipid bilayer vesicles that encapsulate antigenic proteins, protecting them from degradation while enabling controlled release. The nanocomposite microparticles represent solid structures formed through liposome drying, creating a stable powder ideal for mucosal delivery. This approach aimed to create a serotype-independent vaccine capable of protecting against diverse *S. pneumoniae* strains by stimulating pulmonary immunity through intranasal administration. Formulations were produced via microfluidics and converted into NCMPs, which demonstrated excellent stability and protein activity preservation. Female BALB/c mice received intranasal immunization with LP/NCMP suspensions (6 μg total protein). Control groups included saline solution, empty LP/NCMPs (antigen-free), and subcutaneous immunization with purified PspA1, PspA4Pro, or their mixture without nanoparticles. Animals received two doses at 15-day intervals, followed by intranasal challenge 21 days post-boost with *S. pneumoniae* strains ATCC 6303 (ST3, PspA5) or A66.1 (ST3, PspA2). Subcutaneous immunization failed to induce mucosal antibodies, whereas LP/NCMPs generated low but detectable respiratory and vaginal mucosal IgA/IgG. Both individual and combined LP/NCMP formulations demonstrated cross-protection and binding to pneumococcal strains expressing different PspA clades. A balanced IgG1/IgG2a response was observed. Lung-targeted α-GalCer-containing LP/NCMPs induced pulmonary resident memory T cell (CD4^+^ TRM) formation [63]. These results suggest that mucosal immunization with microparticles harboring PspA is a promising approach to reduce pneumococcal pneumonia via induction of protective antibodies.

Roberts et al. progressed to coinfection models simulating more complex clinical scenarios, evaluating the protective potential of PspA as a vaccine antigen in a murine model of secondary *S. pneumoniae* infection following influenza A infection. This strategy was chosen considering that *S. pneumoniae* is one of the main agents of post-influenza complications, with synergy between the two pathogens. The study employed a respiratory coinfection model where BALB/c mice were initially infected with influenza A virus and then challenged intranasally with *S. pneumoniae* during the recovery phase, mimicking the increased susceptibility to bacterial infection observed in humans after influenza. The primary objective was to compare the coinfection effect in mice previously immunized with Prevnar (a vaccine containing capsular polysaccharides from 7 or 13 *S. pneumoniae* serotypes) or with PspA (family 1, clade 2), using the complete recombinant PspA protein. The mice were vaccinated intramuscularly, received a booster 3 weeks after administration, and antibodies were extracted at week 4. Two weeks post-vaccination, the animals were intranasally infected with 10-15 PFU of H1N1 strain A/Puerto Rico/8/1934 (PR8). Body weight was monitored daily, and between days 8–10 post-viral infection—when mice began regaining weight—they were challenged with 1.5 × 10^4^ CFU of *S. pneumoniae* type 2 (strain D39) or with 5 × 10^2^, 5 × 10^3^, or 5 × 10^4^ CFU of *S. pneumoniae* type 3 (strain A66.1), also via intranasal administration. The results indicate that PspA immunization induced higher IgG levels compared to mice vaccinated with Prevnar or unvaccinated controls. It was also observed that protection conferred by both vaccines depended on the bacterial dose used for challenge. At a concentration of 20 × LD_50_ of strain A66.1, all vaccinated mice were protected. PspA immunization also resulted in significant reduction in bacterial load in bronchoalveolar lavage and lung tissue, along with complete bacterial clearance in blood. Finally, unlike Prevnar, PspA conferred protection against *S. pneumoniae* D39, a serotype not included in the conjugate vaccine formulation at the time of the study [56].

Similarly, Majumder et al. tested a vaccine based on outer membrane vesicles expressing PspA (OMV-PspA), also aimed at preventing pneumococcal infection following influenza. *Yersinia pseudotuberculosis* (YptbS46) containing the Asd pSMV92 plasmid can synthesize the α-helical region of PspA, corresponding to amino acid residues 3 to 285, along with monophosphoryl lipid A as an adjuvant, leading to production of outer membrane vesicles (OMVs) containing high amounts of recombinant protein, designated OMV-PspA. Six-week-old male and female Swiss Webster mice were immunized intramuscularly with OMVs in PBS, with a booster dose after 21 days. As controls, they used rPspA adsorbed to Alhydrogel or PBS alone. Intranasal challenge occurred two times. The first challenge was performed on day 36 after the primary dose, with intranasal infection by H1N1 A/California/04/2009 (CA04); nine days later (day 45), the animals were challenged with *S. pneumoniae* (strain D39). A second, later challenge protocol involved influenza infection on day 196 and *S. pneumoniae* challenge on day 205, using the same strains. The OMV-PspA immunization provided 80% protection against secondary Spn challenge, while rPspA immunization provided only 20% protection, and the OMV-NA and PBS groups showed no protection. Monitoring revealed that in the OMV-PspA immunized group, IL-6 and IL-1β levels at 1 DPV (days post-vaccination) were highest, decreasing from 2 DPV onward, with higher serum CRP levels compared to other groups at 1 DPV. The OMV-PspA vaccine induced high titers of anti-PspA IgG, mainly in mucosa, with no detectable IgA titers. There was also a significant increase in lung PspA-specific CD4^+^ and CD8^+^ T cell populations compared to other groups. Thus, OMV vaccination may provide non-specific protection against influenza viral infection while inducing less lung damage. However, this combination has safety limitations. OMV-PspA caused substantial weight loss and prolonged elevation of alveolar macrophages in mice, requiring further investigation to identify the specific OMV components responsible for this reactogenicity [46].

Complementing the analysis of mucosal vaccine strategies, Ortiz Moyano et al. investigated the use of bacterium-like particles (BLPs) derived from *Corynebacterium pseudodiphtheriticum* strain 090104 (Cp 090104) as adjuvants for pneumococcal vaccines containing either PspA protein or the commercial polysaccharide vaccine Pneumovax^®^ 23 (PPV). The aim was to enhance both mucosal and systemic immune responses. For this purpose, the authors developed two distinct experimental models using three-week-old female Swiss albino mice, a strain selected for its susceptibility to pneumococcal infection. To test primary and secondary infection, different approaches were employed. In the first model, animals received either live Cp 090104 or BLPs intranasally for five consecutive days. On day six, they were nasally challenged with *S. pneumoniae* serotype 6B or 19F to evaluate whether Cp or BLPs alone, without prior immunization, could induce protection against primary infection. In the second model, mice were immunized in a prime–boost regimen with three intranasal doses on days 0, 14, and 28, using either the commercial Pneumovax^®^ 23 vaccine or the recombinant chimeric protein vaccine PSPF (PsaA-Spr1875-PspA-FliC), combined with either Cp 090104 or BLPs. On day 30, Poly(I:C), a TLR3 agonist, was administered to induce viral-like pulmonary inflammation. On day 33, animals were intranasally challenged with *S. pneumoniae* serotype 19F to simulate secondary pneumococcal infection. Key findings showed that both Cp 090104 and derived BLPs stimulated airway innate immunity and served as effective adjuvants for both vaccines. The PSPF formulation, containing fragments of PsaA, Spr1875 and PspA proteins fused to flagellin FliC (as an immunomodulatory adjuvant), induced strong immune responses. The study documented increased pneumococcal-specific IgA and IgG production in bronchoalveolar lavage (BAL) and serum; elevated IFN-γ and IL-4 levels (indicating Th1 and Th2 cell activation); and enhanced antibody deposition in respiratory mucosa, particularly with Cp 090104. Vaccination reduced bacterial lung load in both primary and secondary infections. Thus, mucosal adjuvants with either PPV or PSPF containing PspA proved effective, with the advantage that PspA promoted broader immune responses against more conserved proteins and provided higher serotype coverage [62].

Still focusing on complex respiratory infections, Kramskaya et al. proposed a combined vaccination strategy involving influenza and a chimeric protein containing pneumococcal antigens. The authors studied a combined immunization with live attenuated influenza vaccine (LAIV) and a recombinant pneumococcal chimeric protein (PSPF), composed of PsaA, PspA and Shr1875—three *S. pneumoniae* surface proteins—associated with flagellin, a potent activator of innate immune response. Eight to ten-week-old female mice were immunized in different ways. One group received intranasal inoculation with live influenza vaccine PBS (LAIV), PSPF vaccine containing the recombinant PSPF polypeptide diluted in PBS, mixed LAIV+PSPF vaccine or PBS; the control group received single viral and bacterial infection. The vaccine was repeated after 21 days. On days 53 and 54 of the experiment, animals were challenged with different protocols: one group was initially infected with *S. pneumoniae* (strain 73 of serotype 3) and, 24 h later, with influenza virus A/South Africa/3626/13 (H1N1), configuring secondary influenza infection. Another group received viral infection first, followed by *S. pneumoniae*, representing secondary pneumococcal infection. Both infections were performed intranasally with sublethal pathogen doses. In the pneumonia model, viral and bacterial loads in lungs were analyzed 48 h after influenza infection (i.e., 24 h after bacterial superinfection). Results demonstrated that mice vaccinated with the LAIV+chimeric protein combination showed significant reduction in pulmonary viral load, with virus isolation only at minimal titers. In contrast, groups receiving only chimeric protein or PBS showed high viral load. Similarly, the lowest bacterial lung load was observed in the virus-bacteria vaccinated group, while other groups showed high levels of *S. pneumoniae* in lungs. Furthermore, vaccination with the chimeric protein, alone or combined with LAIV, induced high levels of IgG specific against bacterial components, with the most robust humoral response in the combined formulation group, suggesting a synergistic effect. The LAIV-only vaccinated group showed no significant IgG response against bacterial antigens. Thus, combined immunization prevented severe respiratory infections caused by viral-bacterial coinfection, reducing pulmonary pathogenic load and progression risk [57]. These findings support the potential of a combined vaccination strategy employing LAIV and a chimeric pneumococcal protein to prevent secondary bacterial and viral respiratory infections (or to prevent severe outcomes associated with influenza—*S. pneumoniae* coinfections).

Wiedinger et al. investigated molecular strategies to target PspA to antigen-presenting cells by developing a vaccine that fused the N-terminal region of PspA (family 1, clade 2, comprising 303 amino acids) to the Fc portion of murine IgG2a (IgG2a Fc-PspA). The Fc region represents the constant domain of immunoglobulins responsible for interactions with immune receptors (FcγR). Specifically, the IgG2a isotype exhibits high affinity to Fcγ receptors (FcγRI and FcγRIII), promoting enhanced uptake by antigen-presenting cells (APCs) such as macrophages and dendritic cells. This approach aimed at boosting immune responses by facilitating antigen internalization and presentation. Fcγ receptors (FcγR) serve as primary regulators of IgG effector functions in vivo, controlling critical processes including antigen presentation, antibody-dependent cellular cytotoxicity (ADCC), phagocytosis, and activation/proliferation of myeloid cells. The strategy focused on delivering PspA to APCs via FcγR, leveraging the distinct immunomodulatory properties of different IgG subtypes. For immunization, mice were divided into groups of five and administered either 20 µL of PBS, 10 µg of IgG1 Fc or IgG2a Fc fusion proteins, or 5 µg of PspA alone. All groups received booster immunizations on days 14 and 28. Two weeks after the final dose, animals were challenged with 8 × 10^6^ colony-forming units (CFU) of *S. pneumoniae* strain A66.1 and monitored for 21 days. Results demonstrated that immunization with IgG2a Fc-PspA induced significantly higher levels of PspA-specific antibodies across all evaluated isotypes, including IgG and IgA. In contrast, fusion with IgG1 Fc failed to enhance PspA immunogenicity compared to the protein alone. The IgG2a Fc-PspA-induced immune response showed a predominant Th1 profile, with elevated concentrations of IL-2, IFN-γ, and TNF-α in mouse lungs. Additionally, researchers observed increased numbers and frequency of AM1 alveolar macrophages, characterized by a pro-inflammatory phenotype and production of IL-12, IL-23, and nitric oxide. Functionally, bacterial load in lungs was significantly lower in the IgG2a Fc-PspA group at 24 h post-challenge, while groups immunized with PspA alone or IgG1 Fc-PspA showed high pulmonary bacterial loads comparable to controls. These findings reinforce PspA’s central role as a vaccine antigen and demonstrate how antigen presentation format and the specific IgG isotype used for fusion directly influence the magnitude and quality of protective immune responses. Thus, the Fcγ receptor-targeting strategy—particularly through IgG2a—proved especially promising to promote protective Th1-based immune responses [58].

An innovative approach using a nanogel-based trivalent PspA nasal vaccine delivered through a nasal spray has been evaluated in a non-human primate model of pulmonary infection [25]. The vaccine included three PspAs of families 1 and 2, expressed via a *Corynebacterium glutamicum* system. The use of a cationic cholesteryl pullulan nanogel (cCHP) favors adhesion to the nasal mucosa, promoting strong immune activation at the nasal tisuse. The animals received the vaccines 5 times in two weeks intervals, under ketamine anesthesia. Immunization led to the production of serum and lung IgG, and mucosal IgA. Antibodies induced complement C3 deposition on the pneumococcal surface in vitro, and the immunized macaques were protected from intratracheal infection with two pneumococcal strains expressing family 1 and family 2 PspAs, attesting the efficacy of this mucosal vaccination to prevent pneumonia.

In addition to mucosal strategies, several subcutaneously administered vaccine formulations have been proposed, particularly those based on fusion proteins combining PspA with other pneumococcal antigens [41,44,60,94]. Santos et al. evaluated the immune response and protection induced by a vaccine composed of a fusion protein between PspA (pneumococcal surface protein A) and a detoxified derivative of pneumolysin (PlD1) in a murine model of lobar pneumococcal pneumonia. The chimeric construct used the N-terminal region of PspA from a serotype 14 strain (St 245/00), classified as PspA1 (family 1), containing predominantly the α-helical domain (αHD), which harbors the main immunogenic epitopes of the protein. Female BALB/c mice were immunized subcutaneously with three doses of the chimeric protein rPspA1-PlD1, administered at 14-day intervals, using Al(OH)_3_ as an adjuvant. The control group received only the adjuvant. Two weeks after the last immunization, the animals were challenged intranasally with *S. pneumoniae* serotypes 14 (St 245/00) and 19F (P854), both considered to have low invasiveness, while colonizing the lungs and replicating the hallmarks of lobar pneumonia. Seven days after challenge, pulmonary bacterial loads, cytokine levels in the bronchoalveolar lavage fluid (BALF), and airway inflammation were assessed. The results showed that the rPspA1-PlD1 vaccine conferred significant protection against pneumococcal pneumonia, associated with an early and controlled local inflammatory response. There was a significant increase in TNF-α and IL-6 levels in BALF, with rapid mobilization of immune cells within 6 h of infection, peaking at 12 h, and followed by a marked reduction at 24 h. Lung integrity was preserved in immunized mice, which showed only mild leukocytic infiltration, whereas control animals presented moderate inflammation, congestion, and alveolar hyperplasia. Pulmonary bacterial loads in vaccinated animals remained very low throughout the experiment. Furthermore, antibodies generated by the vaccine recognized heterologous *S. pneumoniae* strains, suggesting potential for cross-protection. The study concluded that the rPspA1-PlD1 formulation induced opsonophagocytic antibodies, promoting a balanced local inflammatory response, and providing protection against both pulmonary and systemic infection, with higher survival rates in immunized mice compared to the control group [60].

Yokota et al. adopted a distinct strategy, applying a prime–boost approach with different immunization routes using fragments from the N-terminal region of PspA. The formulation employed an intramuscular priming followed by a mucosal booster, aiming to simultaneously stimulate systemic and mucosal, immune responses. In mice, the booster was administered intranasally, whereas in monkeys the booster was delivered intratracheally aiming to simultaneously stimulate systemic and mucosal immune responses. In BALB/c mice, the PspA3+2 protein was emulsified in a water-in-oil-in-water (WOW) system and adjuvanted with curdlan (a β-glucan derived from bacteria) and CpG-ODN. Immunization induced the production of serum IgG and secretory IgA (SIgA) in bronchoalveolar lavage fluid (BALF) and conferred significant protection against respiratory infection caused by *S. pneumoniae* serotype 6A (family 1, clade 2) compared with control groups. The preclinical model also included *Macaca fascicularis* monkeys, which, after the prime–boost regimen with PspA3+2/WOW adjuvanted with curdlan alone or in combination with CpG-ODN, exhibited detectable levels of lung-specific SIgA, indicating the potential of this formulation to elicit effective immune responses in non-human primates [61].

Building on the rBCG-PspA-PdT strategy previously described in neonatal sepsis models, Goulart et al. evaluated the protective potential of the formulation in a murine aspiration pneumonia model. Female C57BL/6 mice were immunized subcutaneously with a primary dose of the recombinant BCG expressing the chimeric protein, followed 30 days later by a booster dose of recombinant PspA–PdT (rPspA–PdT) adsorbed into Al(OH)_3_. Control groups received wild-type BCG (WT-BCG), rPspA–PdT alone, or saline. To assess the immune response, animals underwent intranasal instillation with *S. pneumoniae* serotype 3 (strain WU2, PspA^+^) in a murine aspiration pneumonia model. The heterologous immunization regimen (rBCG PspA–PdT/rPspA–PdT) promoted early bacterial clearance from the lungs, with reduced bacterial loads in bronchoalveolar lavage fluid (BALF) as early as 12 h post-challenge, and no detectable bacteria after 48 h. The vaccination scheme induced both humoral and cellular immune responses, including increased production of anti-PspA IgG, greater C3 deposition on the bacterial surface, reduced neutrophil influx, and lower inflammatory cytokine levels, while preventing tissue damage and improving animal survival [59]. These findings reinforce the efficacy of BCG as an effective antigen delivery platform for vaccines targeting respiratory pathogens with the induction of robust antibody and cellular responses.

Collectively, the evidence from different models demonstrates a central role for PspA in protection against pneumococcal pneumonia. PspA-based formulations—whether delivered via nanocomposite microparticles, outer membrane vesicles (OMVs), or chimeric fusion proteins—promote protective responses that contribute to pneumococcal clearance. The strategic use of innovative delivery systems and adjuvants, such as recombinant BCG platforms, potentiates both the antibody and cellular defenses, including lung-resident memory T cells (TRM).

Furthermore, the success of these vaccines in complex co-infection scenarios (such as post-influenza pneumonia) highlights their clinical relevance in reducing the global burden of secondary bacterial infections. Because PspA induces cross-protection across diverse serotypes and clades, it represents a pivotal shift toward a serotype-independent strategy. Ultimately, these findings suggest that integrating PspA into multifaceted vaccine schemes—particularly those targeting the respiratory mucosa—offers a promising strategy for preventing severe pneumonia, reducing hospital costs, and overcoming the limitations of current capsular-based immunizations.

## 4. Otitis

Otitis media (OM) is inflammation of the middle ear, most common in children, associated with infectious, allergic, anatomical, genetic, and environmental factors. *S. pneumoniae* represents the primary bacterial pathogen involved in this disease [95,96]. The acute form of otitis (AOM) typically occurs following upper respiratory tract infections (URTIs) and causes fever, otorrhea, irritability, and in severe cases, hearing loss. Otitis media with effusion (OME) is characterized by non-purulent fluid behind the tympanic membrane without pain, while the chronic form (COM) involves pain, persistent discharge, and hearing impairment. In the U.S., annual OM-related costs exceed $5 billion, and globally, the disease accounts for approximately 28,000 deaths and nearly half of permanent hearing loss cases [97,98]. Multiple studies have investigated PspA’s role in OM pathogenesis and its potential as a vaccine target, employing distinct experimental models including chinchillas, rats, and mice, along with in vitro approaches [64,65,66,67,68,69], as summarized in Table 3.

The study published by Schachern et al. employed an experimental model involving bilateral otitis media induction in adult chinchillas, using *S. pneumoniae* strain D39 (serotype 2, NCTC 7466) to evaluate the role of surface proteins PspA and PspC in virulence. Four experimental groups were bilaterally inoculated in the middle ear with: (1) Wild-type D39 (with intact PspA and pspC genes); (2) PspA^−^ mutant (lacking PspA gene); (3) PspC^−^ mutant (lacking pspC gene); and (4) PspA^−^/PspC^−^ double mutant (deficient in both proteins). Inoculation was performed via intrabullar injection (directly into the tympanic bulla) with 0.5 mL bacterial suspension per middle ear under ketamine hydrochloride/acepromazine maleate anesthesia. Forty-eight hours after inoculation, animals were euthanized for sample collection: middle ear effusions for bacterial colony-forming unit (CFU) counts; cochleae and round window membranes (RWM) for histological analysis including quantification of inflammatory infiltrate (polymorphonuclear and mononuclear cells) in toluidine blue-stained sections examined by light microscopy. Effusions showed high bacterial loads in wild-type and PspC^−^ groups, while PspA^−^ and double mutant groups had undetectable bacteria. Histologically, wild-type and PspC^−^ groups exhibited intense inflammatory infiltration, whereas PspA-deficient groups showed reduced inflammatory responses. These findings demonstrate PspA’s critical role in pneumococcal otitis media [65].

Complementing those results, a study compared a wild-type *Streptococcus pneumoniae* strain with isogenic mutants lacking PspA (PspA^−^), pneumolysin (ply^−^), or both proteins (ply^−^/PspA^−^) in a chinchilla otitis media model. Loss of PspA alone resulted in rapid bacterial clearance from the middle ear, with no viable bacteria detected at latter time points. This effect was attributed to reduced viability in vivo, as the PspA^−^ mutant showed normal growth in vitro but increased susceptibility to host defense mechanisms in the middle ear. In contrast, the ply^−^/PspA^−^ double mutant displayed a distinct phenotype, with detectable bacterial growth despite reduced tissue damage and inflammation, indicating that the combined deletion of pneumolysin and PspA results in a non-additive virulence profile [68].

Schachern et al. also investigated how the absence of PspA, PsaA, and pneumolysin affects the structure and permeability of the round window membrane (RWM)—the primary interface between middle and inner ear. PspA deletion resulted in markedly reduced virulence: only one animal died, and surviving subjects showed no bacteria in either RWM or scala tympani. Inflammation was limited to the RWM epithelium in just two animals. These findings demonstrate PspA’s direct contribution to pneumococcal penetration into cochlear structures, underscoring its role in OM progression to deeper infections [24].

A study by Tsuprun et al. investigated the role of PspA protein inducing sensorineural hearing loss associated with the disease. The research was based on the observation that, although OM is generally linked to conductive hearing loss, there is growing evidence that bacterial products and inflammatory mediators can cross the round window membrane, reach the inner ear, and cause cochlear damage. To explore this hypothesis, healthy chinchillas were inoculated in the tympanic bulla with either *S. pneumoniae* serotype 2 (wild-type D39 strain) or isogenic strains deficient in pneumolysin or PspA, as well as a control group that received only phosphate-buffered saline. Each animal received 0.5 mL of the bacterial suspension, and auditory function was assessed before and 28 days after infection using auditory brainstem responses (ABRs) at frequencies from 1 to 32 kHz, followed by histopathological analysis of the cochlea. The results showed that infection with the PspA^−^ strain completely prevented sensorineural hearing loss and caused no cochlear damage, even when administered at a higher dose than the wild-type strain. In contrast, the D39 strain caused a significant elevation in hearing thresholds and structural alterations in the cochlea. These findings highlight the contribution of PspA to OM pathogenesis and its relevance as a promising immunogen for vaccine development aimed at preventing hearing-related complications [66].

In summary, the critical role of PspA in pneumococcal otitis media is defined by its contribution to bacterial survival and tissue invasion, affecting the structural integrity of the infection site. Mechanistically, PspA serves as a primary defense against the host complement system, preventing C3 deposition and subsequent phagocytic clearance by the host [24]. These findings align with previous observations that PspA deficiency leads to rapid in vivo clearance due to increased susceptibility to middle ear defenses [68]. Furthermore, PspA expression contributes to the breakdown of the round window membrane barrier; its absence limits inflammation to the epithelium and prevents the progression to sensorineural hearing loss by blocking bacterial access to the cochlear structures [65,66].

The protective efficacy of PspA immunization against pneumococcal AOM was evaluated by Habets et al. in a mouse model of coinfection with influenza A (H3N2 strain) followed by *S. pneumoniae* (serotype 19F) infection—a clinically relevant condition often associated with the progression of pneumococcal colonization to middle ear infection. Female mice aged 6 to 8 weeks from BALB/c, C57BL/6 (B6), B6.μMT^−/−^ (antibody-deficient), and B6.IL17RA^−/−^ (lacking the IL-17 receptor) strains were used. Animals were immunized intranasally with three doses of purified rPspA (5 µg per dose), obtained from the *S. pneumoniae* TIGR4 strain. The formulation was administered with or without the adjuvant cholera toxin subunit B (CTB). Three weeks after the final immunization, mice were challenged intranasally with influenza A virus (strain A/Udorn/307/72—H3N2). Three days after viral infection—allowing sufficient time to simulate virus-induced respiratory mucosal dysfunction—animals were infected intranasally with 10^4^ colony-forming units (CFU) of *S. pneumoniae* BHN100 strain (serotype 19F). Three days later, mice were euthanized for quantification of bacterial load in nasal washes and middle ear homogenates. Intranasal vaccination with recombinant PspA co-administered with the CTB adjuvant conferred significant protection against pneumococcal OM, reducing bacterial loads in the middle ear. Protection was associated with the induction of local IgA and IgG antibodies and dependence on IL-17-mediated signaling. In contrast, administration of PspA alone did not confer protection, highlighting the importance of immune co-stimulation for vaccine efficacy. These results reinforce the potential of PspA as a vaccine antigen against otitis media, particularly when combined with adjuvants that stimulate mucosal antibody and cellular immune responses [69].

Similarly, a study aimed to investigate the efficacy of active immunization with PspA in preventing AOM, using an experimental model in adult Sprague-Dawley rats immunized subcutaneously with three doses of PspA formulated in Freund’s adjuvant. Infection was induced by direct inoculation of 5 × 10^6^ CFU of a serotype 6A strain into the middle ear cavity with a bacterial suspension, following surgical exposure of the tympanic bulla. Infection was monitored by otomicroscopy, with OM defined as the presence of purulent exudate in at least one evaluation. Active immunization with PspA conferred protection against purulent otitis media in rats challenged with *S. pneumoniae*. None of the immunized animals developed infection, in contrast to the control groups, which showed a high incidence of otitis. Protection was associated with the induction of high levels of specific IgG, indicating that the humoral response generated by PspA was sufficient to prevent middle ear infection. These findings further highlight the efficacy of PspA as a vaccine candidate against pneumococcal otitis media [64].

Finally, Li-Korotky et al. employed an in vitro model using human middle ear epithelial cells to evaluate the differential expression of PspA in opaque (O) and transparent (T) variants of *S. pneumoniae*, focusing on their adaptation and virulence during middle ear infections. These variants represent morphologically distinct bacterial forms: opaque variants exhibit higher expression of polysaccharide capsule, conferring greater resistance to opsonophagocytosis and being commonly associated with invasive infections such as sepsis. In contrast, transparent variants express less capsule but higher amounts of surface proteins, such as PspA; they display greater adhesion to epithelial cells and predominate in mucosal colonization. The *S. pneumoniae* strains used were derived from human clinical isolates, which present distinct gene expression patterns and colony morphologies. To explore the behavior of these variants under conditions that mimic the inflammatory environment of otitis media, human middle ear epithelial cells were exposed to pneumococci under conditions simulating physiological dysfunctions of the middle ear, such as Eustachian tube obstruction (ETO) and the presence of tympanostomy tubes (TT). This model allowed the assessment of differential expression levels of virulence genes in response to the pathological microenvironment. PspA was analyzed exclusively as an endogenous virulence factor, with emphasis on its differential expression among phenotypic variants. The study demonstrated that PspA expression was significantly higher in the transparent (T) variants of *S. pneumoniae*, both under basal conditions and after epithelial adhesion. In the in vitro human middle ear epithelial cell model, PspA gene expression was significantly upregulated in T variants adhered to the epithelium under simulated tympanostomy tube (TT) conditions, compared to opaque (O) variants. These findings indicate that PspA is strongly associated with the phenotype most adapted to colonization and persistence in the middle ear mucosa—key traits for the development of otitis media. The differential expression profile of PspA, combined with its well-known role in immune evasion, further supports its relevance as a promising vaccine candidate, particularly for the prevention of non-invasive pneumococcal infections such as otitis media, where adhesion and interaction with the epithelium are critical events in pathogenesis [67].

Altogether, these studies demonstrate that PspA plays a critical role in the pathogenesis of otitis media by promoting bacterial persistence, middle ear inflammation, and subsequent cochlear damage. However, the implementation of PspA as a protective immunogen relies on the adjuvant selection. Systemic adjuvants (e.g., Freund’s) can promote protection by inducing high titers of circulating IgG that transudate into the middle ear to neutralize pathogens. Nevertheless, for human application—where acute otitis media (AOM) is typically preceded by viral respiratory stress—a mucosal approach appears more strategic. As demonstrated in co-infection models, the use of mucosal adjuvants like Cholera Toxin subunit B (CTB) is critical for coordinating a dual-layered defense: the induction of secretory IgA to halt initial attachment and the activation of IL-17 signaling to recruit neutrophils for bacterial clearance [69]. By targeting this IL-17/IgA axis, PspA-based vaccines can limit pneumococcal progression at the nasopharynx, thereby preventing the inflammatory cascade that leads to RWM permeability and tissue damage [65]. Ultimately, integrating PspA with clinically viable mucosal adjuvants offers a biologically tailored alternative to current conjugate vaccines, specifically designed to reduce the complex disease burden of pediatric AOM.

An important aspect that should be considered when comparing the results from different studies is the choice of the experimental model, which significantly influences the aspects of OM pathogenesis being explored. While murine models provide deep insights into the systemic immune response to PspA-based vaccines, the chinchilla model remains a gold standard for studying localized complications such as round window membrane damage and sensorineural hearing loss, due to its large, accessible tympanic bulla and an auditory system that closely mimics human anatomy [66,68]. However, this model has important limitations, such as high cost, specialized housing requirements, and a more limited set of available immunological reagents like antibodies, compared to murine models. Conversely, in vitro models serve to isolate the molecular mechanisms of PspA-mediated evasion but lack the complex “milieu” of the middle ear.

## 5. Colonization

Pneumococcal colonization of the nasopharynx represents an initial and crucial step in invasive pneumococcal disease pathogenesis, influenced by carriage dynamics and competition between different serotypes [53]. *S. pneumoniae* frequently colonizes the human nasopharynx asymptomatically but can also cause diseases including sinusitis, otitis media, pneumonia, sepsis, and meningitis. High carriage rates have been reported among children, particularly in low-income countries, where colonization in children under five years ranges from 20% to 93.4% [99]. Furthermore, concurrent carriage of two or more pneumococcal serotypes is common, reaching up to 50% of carriers [53,99,100]. Therefore, understanding interactions between different serotypes and PspA-induced immune responses is essential for developing effective vaccine strategies against colonization. Studies investigating the effect of PspA-based vaccines on pneumococcal colonization are listed in Table 4.

Kuipers et al. [72] evaluated the impact of genetic background across different mouse strains on nasal colonization by *S. pneumoniae* and the efficacy of a PspA-based vaccine in this process. Seven-week-old female C57BL/6J, BALB/c, and CB6F1 (BALB/c × C57BL/6J hybrids) mice were administered intranasally with 10 μg of recombinant PspA from TIGR4 using 4 μg of cholera toxin B subunit (CTB) as adjuvant. The scheme included three vaccine doses at two weeks intervals between each immunization. Three weeks post-final immunization, mice were intranasally challenged with 10^6^ CFU of strain TIGR4, and colonization was assessed five days later by counting CFU recovered from nasal washes. Among controls (CTB alone), BALB/c mice showed higher colonization density than C57BL/6 and CB6F1. PspA vaccination significantly reduced colonization across all strains, with the greatest reduction in CB6F1 hybrids where nearly all vaccinated animals showed undetectable colonization. Intra-group variation was lowest in BALB/c, while CB6F1 and C57BL/6 exhibited wider bacterial count dispersion. These findings indicate that while intranasal CTB-adjuvanted PspA provides protection across genetic backgrounds, the magnitude of colonization protection varies by host strain. The authors suggest CB6F1’s enhanced resistance may stem from combined Th1 (C57BL/6-dominant) and Th2 (BALB/c-dominant) immune responses, yielding a more balanced cellular/humoral immunity that more effectively reduces pneumococcal load [72]. This result demonstrates PspA’s ability to prevent pneumococcal colonization in mice, while highlighting the importance of a balanced Th1/Th2 response to protection.

Conjugate vaccines including PspA fused to polysaccharides have also been tested in murine models of colonization. The work from Kaplonek et al. evaluated the protective efficacy of PspA4Pro conjugated to capsular polysaccharide serotype 14 (forming PS14-mPspA4Pro conjugate) against nasal colonization by *S. pneumoniae*. Female BALB/c mice were immunized subcutaneously with three doses (days 0, 14, 28) of PS14-mPspA4Pro conjugate or the co-administered unconjugated antigens. Control mice received Alum diluted in PBS. On day 42, blood was collected for serological analysis, followed by intranasal challenge with 1 × 10^7^ CFU of *S. pneumoniae* strain 0603 (serotype 6B, clade 1). Nasal washes were collected seven days post-challenge for bacterial counting. Both conjugate-vaccinated and co-administered antigen groups showed significant reduction in nasal colonization (100-fold to 1,000-fold) when compared to controls. The protective efficacy was similar between vaccinated groups, demonstrating that PspA4Pro conferred cross-clade protection against colonization with a heterologous strain. These findings support PspA’s potential as a vaccine antigen capable of cross-clade protection, whether free or conjugated to polysaccharide [75].

Carneiro et al. investigated the protective efficacy of *S. pneumoniae* extracellular vesicles (pEVs) against nasal colonization and invasive infection. pEVs are membrane-derived structures secreted into extracellular space, carrying selective cargo including key pneumococcal virulence antigens such as pneumolysin (Ply), maltose/maltodextrin ABC transporter (MalX), chaperone PrsA, pneumococcal surface protein C (PspC), and pneumococcal surface protein A (PspA), capable of inducing immune responses when administered to hosts. Specific pathogen-free (SPF) female BALB/c and C57BL/6 mice were subcutaneously immunized with 1 μg, 2.5 μg, or 5 μg of pEVs derived from strain R6 (WT pEVs) or R6 ΔPspA (ΔPspA pEVs—PspA-deficient), diluted in 100 μL saline. Control groups received saline alone. Three doses were administered at 15-day intervals. For intranasal immunization, mice received three doses of 750 ng WT or ΔPspA pEVs in 20 μL saline following the same schedule. Challenges were performed 21 days post-final immunization for both pneumonia and colonization models. For nasal colonization challenges, C57BL/6 mice were inoculated with 10 μL containing 1 × 10^6^ CFU of strain 0603 (ST6B, PspA1). pEVs demonstrated significant protection against nasal colonization, even across different capsular types. Similar levels of protection were observed in mice immunized with WT and ΔPspA pEVs, suggesting that PspA is not essential for preventing nasal colonization in this model. Post-colonization assays revealed that intranasal immunization with R6 pEVs protected mice against pneumococcal nasal colonization despite low circulating antibody titers, indicating that high antibody levels are not required for this protection [76].

The importance of vaccine strategies in preventing co-colonization has also been addressed in studies combining PspA with other components. Colichio et al. established a murine model of nasopharyngeal co-colonization using combinations of vaccine-type (VT) and non-vaccine-type (NVT) *S. pneumoniae* strains, genetically marked with erythromycin (erm) or spectinomycin (spec) resistance through iga insertion without affecting virulence in mice. The vaccine formulation consisted of recombinant PspA proteins from clades 1 and 4 (PspA1+PspA4) combined with whole-cell pertussis vaccine (wP) as adjuvant, administered intranasally in three 10 μg doses at two-week intervals. PCV13 and PCV13 combined with PspA1+PspA4+wP were also evaluated. Five days after intranasal challenge with 5 × 10^5^ CFU of each strain, colonization was quantified by selective plating of nasal washes. In the VT4+NVT33 model, PCV13 significantly reduced VT4 but showed a trend toward increased NVT33 colonization, whereas PspA1+PspA4+wP reduced both strains, achieving significant reduction (of two logs) of NVT33 compared to wP alone. The combination PspA1+PspA4+wP+PCV13 reduced both strains compared to PCV13 alone. In the VT23F+NVT15B/C model, PCV13 did not reduce VT23F (PspA2), consistent with lack of serological recognition, while PspA1+PspA4+wP—either alone or combined with PCV13—significantly reduced both strains. The wP adjuvant alone also showed protective effects, though with varying efficacy between strains. These results indicate that immunization with PspA1+PspA4+wP, either alone or combined with PCV13, provides broader protection against co-colonization by both VT and NVT strains compared to PCV13 alone, without promoting serotype replacement [74]. This result suggests a protective role for PspA in controlling concomitant infections with different pneumococcal strains—a common scenario especially in children.

In another study, Tostes et al. compared different combinations of PspA from clades 1, 2, 3, and 4 associated with the whole-cell pertussis vaccine (wP) in protection against pneumococcal co-colonization. Four *S. pneumoniae* strains were used: St491/00 (serotype 6B, PspA1) and St472/96 (serotype 6B, PspA4, trimethoprim-resistant), as well as SPEC 6B (serotype 6B, PspA3, spectinomycin-resistant) and EMC 23F (serotype 23F, PspA2). The vaccination strategy consisted of intranasal immunization of five- to seven-week-old female specific-pathogen-free C57BL/6 mice with 5 μg of recombinant PspA protein combined with 1/8 of the wP dose, administered in two doses 14 days apart; in groups receiving a mixture of two proteins, each was given at 2.5 μg. Mice were anesthetized intraperitoneally with 200 μL of a solution containing 0.2% xylazine and 0.5% ketamine before immunization. Three weeks after the final dose, the animals were anesthetized again with the same solution and challenged intranasally with a mixture of two pneumococcal strains (5 × 10^5^ CFU of each strain), inoculated into both nostrils. Five days post-challenge, the mice were euthanized intraperitoneally with a lethal dose of xylazine/ketamine, and nasal colonization was assessed by plating nasal washes on blood agar with or without selective antibiotics, enabling differential quantification of each strain in the co-colonization model. The results showed that the formulations rPspA1+wP, rPspA3+wP, and rPspA4+wP significantly reduced colonization by both serotype 6B strains compared to the saline group, with rPspA1+wP and rPspA4+wP showing additional reductions against their homologous strains compared to the wP group. Combined strategies (rPspA1+rPspA4+wP or a heterologous regimen of rPspA1/wP followed by rPspA4/wP) promoted greater reductions in colonization by both strains than immunization with a single antigen. In the challenge with 23F OPKA (PspA2) and 6B OPKA (PspA3), rPspA1+wP reduced colonization by 23F OPKA, whereas rPspA4+wP reduced colonization by both strains compared to saline [71].

Following the hybrid protein approach, Converso et al. [35] analyzed a chimeric protein combining PspA fused to PotD and its potential for protection against invasive pneumococcal infection and nasopharyngeal colonization in mice. PotD, like PspA, is a membrane protein of *S. pneumoniae* capable of binding polyamines and transporting them from the extracellular medium into the cytoplasm. For immunization, a hybrid protein was generated through the genetic fusion of the gene fragment encoding the N-terminal region plus the proline-rich region of PspA from strain St P490 with the potD gene from strain St 540/99, followed by PCR amplification. Animals were immunized subcutaneously with three doses of 10 µg rPspA, 12 µg rPotD, or 22 µg rPspA-PotD at 14-day intervals, using 0.9% sterile saline with 100 µg Al(OH)_3_ as an adjuvant. The adjuvant alone diluted in saline was used as a control. Fourteen days after the last immunization, two challenges were performed. In the colonization challenge, animals were inoculated into one nostril with 1 × 10^7^ CFU of the noninvasive pneumococcal strain St 0603 diluted in 10 µL of sterile PBS. Results demonstrated that the inclusion of PotD reduced nasopharyngeal colonization, an effect not previously observed with subcutaneous immunization containing PspA alone. Immunization with the chimeric protein led to production of antibodies with increased binding capacity to pneumococcal strains of various serotypes and genetic backgrounds, enhanced opsonophagocytosis, and IL-17 secretion by splenocytes. The rPspA-PotD hybrid was highly immunogenic and induced stronger IgG responses than the individual proteins, reducing nasopharyngeal colonization, which decreases transmission and prevents disease, thus demonstrating the potential and efficacy of the PspA-PotD fusion in hampering disease progression [35].

Goulart et al. [101] used recombinant *M. bovis* BCG strains expressing pneumococcal antigens, including PspA-PdT, in a prime–boost regimen against nasal colonization by *S. pneumoniae*. C57BL/6 mice were immunized in a prime–boost scheme, in which the first dose consisted of recombinant BCG expressing SP 0148, SP 2108, or the fusion protein PspA-PdT (PspA with broad cross-reactivity+detoxified pneumolysin), followed by a boost with the corresponding purified recombinant protein; the combination of the three rBCG strains (rBCG Mix) followed by the three proteins (rMix) was also tested. The challenge strain used was *S. pneumoniae* St 603 (serotype 6B), administered intranasally three weeks after the last vaccine dose. Seven days post-challenge, colonization was assessed by retrograde nasal wash, with CFU counting on blood agar. The rBCG 0148/rSP 0148 and rBCG 2108/rSP 2108 regimens induced high levels of IL-17A and IFN-γ after in vitro stimulation and significantly reduced nasal colonization compared to the saline and WT-BCG/rMix groups. The rBCG PspA-PdT/rPspA-PdT formulation showed no significant effect on colonization, whereas the rBCG Mix/rMix combination maintained the protection observed for SP 0148 and SP 2108 individually. These results indicate that SP 0148 and SP 2108, when delivered by rBCG, elicit robust Th17 responses and confer protection against pneumococcal colonization by serotype 6B, whereas the PspA-PdT fusion protein, under the tested conditions, did not demonstrate the same protective effect [43]. These results may be explained by a lower activation of protective Th17 responses by PspA-PdT; while these proteins are able to promote string antibodies responses that can protect against invasive infection, previous data suggests that Th17 is a key factor in protecting against colonization [101].

Kuipers et al. [73] used laboratory strains and human clinical isolates of *S. pneumoniae*, including the TIGR4 strain and strains from the Pneumococcal Bacteraemia Collection Nijmegen (PBCN), to evaluate a murine model of nasal colonization. The vaccine formulation was based on outer membrane vesicles (OMVs) from attenuated *Salmonella typhimurium* (SL3261ΔtolRAΔmsbB) displaying on their surface the α1α2 fragment of PspA derived from TIGR4. This region, located between the signal sequence and the proline-rich region (PRR), has been identified as responsible for inducing Th17-mediated protection. Mice were intranasally immunized with OMVs containing PspA α1α2 or with control OMVs (lacking antigen) and subsequently challenged intranasally with 10^6^ CFU of pneumococcus in 10 μL of PBS, using either the homologous TIGR4 strain or heterologous strains selected based on their in vitro IL-17A induction profile (“high IL-17A” or “low IL-17A”) determined in an ex vivo assay. Colonization challenge was assessed by counting CFUs recovered from nasal washes. Results showed that vaccination with PspA α1α2-OMVs conferred significant protection against colonization by the homologous strain and by heterologous strains from the “high IL-17A” group, but not against those from the “low IL-17A” group. This protection correlated with elevated IL-17A levels in the nasal mucosa, demonstrating that the Th17 response induced by the α1α2 fragment is critical for protection, and that the ex vivo IL-17A assay may predict vaccine efficacy in reducing pneumococcal colonization [73].

Finally, Fukuyama et al. investigated the safety and immune activation of a cCHP nanogel containing a family 1 PspA fragment, in non-human primates [70]. Rhesus macaques were immunized intranasally with 5 doses of cCHP-PspA in two-week intervals. The vaccine induced both local and systemic antibody responses, while antisera from immunized animals conferred passive protection in mice against intravenous challenge with heterologous strains, confirming robust functional activity and broad cross-protection. At the cellular level, immunization elicited significant increases in IL-4 and IL-17 production by CD4+ T cells, alongside elevated levels of antigen-specific IgG1, characterizing a balanced Th2 and Th17 immune response. Furthermore, the study identified the upregulation of immunologically relevant microRNAs, specifically miR-181a and miR-326, within the serum and respiratory tissues; these findings suggest a regulatory association between these miRNAs and both lymphocyte differentiation and the observed Th17-mediated response [70].

Collectively, while most studies demonstrate a role for PspA in preventing *S. pneumoniae* colonization in different experimental models, the observation that multiprotein vesicles lacking PspA show comparative protection to those harboring this antigen suggest that PspA is not an absolute requirement for protection in this niche. Furthermore, PspA alone and PspA-PdT fusion vaccines fail to confer protection in different studies. Thus, the protective efficacy of PspA against colonization seems to be highly dependent on the use of strong mucosal adjuvant platforms like wP and mainly, the combination with other proteins, in multicomponent or vesicle-based formulations.

A central protective mechanism in pneumococcal vaccines that impact colonization involves the induction of strong inflammatory responses, specifically Th17, with a balanced Th1/Th2 profile also showing protection in a few studies. Th17 cells are the specialized “sentinels” responsible for recruiting neutrophils and macrophages to kill clearance the bacteria from the mucosal surfaces [101]. Meanwhile, low antibody levels did not affect the protective efficacy of protein-based vaccines, suggesting that antibody production is not a key mechanism in preventing pneumococcal colonization.

## 6. Conclusions

PspA-based vaccine formulations, whether used in isolation, as fusion proteins, in combination with other antigens, delivered via live or particulate vectors, or associated with mucosal adjuvants, have demonstrated reduced bacterial burden, increased survival, and induction of functionally relevant humoral and cellular responses in different contexts. Immunization with PspA provides strong protection against invasive diseases (sepsis, pneumonia, otitis media), whereas its efficacy in preventing nasopharyngeal colonization is limited and requires adjuvants or combination with other antigens. Evidence from phase I clinical trials further supports its initial immunogenicity and safety [26,27,28]. A limitation that must be highlighted is the antigenic variability among PspA families and clades, reinforcing the need for multivalent formulations. Several studies have included components from families 1 and 2 to enhance cross-reactivity; moreover, fragment selection should prioritize epitopes with proven protective efficacy, such as the initial ~100 amino acids of the N-terminal region and elements of the PRR in their native context, such as the NPB, as well as chimeric constructs capable of enhancing complement activation and opsonophagocytic activity. Mucosal strategies, such as systemic+mucosal prime–boost regimens, liposomes/nanoparticles, and the use of specific adjuvants, have shown promise in reducing colonization and transmission, although they require standardization and long-term safety assessment, particularly in the case of OMVs and recombinant BCG.

Recent data from a human trial using combinations of PspA fragments and pneumolysin (namely PBPV) have demonstrated significant potential in overcoming the serotype-limited protection of traditional polysaccharide-conjugate vaccines by eliciting broad, multifunctional antibody response that extends beyond the strains covered by current CPS-based formulations. Findings indicate that while PBPV may exhibit a shorter duration of immunogenicity compared to polysaccharide vaccines, it triggers robust Fc-mediated effector functions, including opsonophagocytic activity (OPA) and antibody-dependent neutrophil phagocytosis (ADNP). These results suggest that targeting conserved antigens like PspA can provide a more universal protective threshold by mediating immune clearance across diverse serotypes, including those currently associated with “serotype replacement” in vaccinated populations [27].

In summary, PspA represents a relevant and versatile vaccine candidate, especially as a component of multivalent protein-based vaccines and/or in combination with polysaccharides, with the potential to broaden serotype-independent protection against multiple types of disease. The use of nanoparticles and/or live vector systems may expand the protective efficacy of PspA, and validation in phase II/III clinical trials will be pivotal to confirm its impact on public health.

## Figures and Tables

**Table 1:** Studies evaluating PspA as a vaccine candidate against pneumococcal sepsis.

Study	Model	Formulation	Results
Full-length PspA and PspA fragments			
Daniels et al., 2010 [16]	CBA/N mice	Recombinant proline-rich (PR) regions of PspA and PspC; passive immunization with anti-PR or anti-NPB monoclonal antibodies	Protection against lethal infection; PR epitopes antibody-accessible on pneumococci; survivors had increased anti-PR antibodies; PR/NPB regions conserved and cross-reactive → potential vaccine targets.
Tamborrini et al., 2015 [29]	NMRI mice	SVLPs displaying B-cell epitopes from the proline-rich region (PRR) of PspA: CCL-PR1; CCL-NPB; CCL-PR1+CCL-NPB; CCL-PR2/N; CCL-PR2/C	SVLPs containing PRR-derived epitopes, especially NPB and the C-terminal PR2 variant, conferred measurable protection against pneumococcal sepsis.
Girgis et al., 2020 [30]	BALB/c mice	Recombinant proline-rich region (PRR) of PspA+Alum adjuvant (active immunization) or anti-PRR polyclonal antibodies (passive immunization)	No survival benefit after lethal challenge (*S. pneumoniae* 19F, 1 × 10^7^ CFU); all animals succumbed; vaccination induced anti-PRR IgG titers.
Multicomponent and chimeric vaccines			
Darrieux et al., 2007 [31]	BALB/c mice	PspA fragments; PspA hybrids PspA1ABC-4B and PspA1ABC-3AB+alum (i.p.)	Cross-protection Increased C3 deposition on the bacterial surface.
Goulart et al., 2013 [32]	BALB/c mice	s.c. recombinant fusion proteins: PspA1-PlD1, PspA1-PlD2, PspA2-PdT (fusions of PspA clades 1 or 2 with detoxified pneumolysin mutants), with alum.	Increased anti-PspA & anti-Ply antibodies; Increased complement deposition; increased OPA (heterologous strains); sera neutralized Ply hemolysis; increased mouse survival; PspA1-PlD1 & PspA1-PlD2 conferred 100% protection.
Piao et al., 2014 [33]	C57BL/6J mice	Fusion proteins PspA2+4, PspA2+5, or PspA3+2+CpG ODN+alum (s.c.)	PspA3+2: significant protection against strains from clades 1–5; sera showed >60% IgG binding to all clades. PspA2+4 and PspA2+5: protection against clades 2, 4, 5, but failed against clades 1 and 3.
	C57BL/6J mice (sera tested)	Same fusion proteins (PspA2+4, PspA2+5, PspA3+2)	Antiserum from PspA3+2 vaccination bound strongly to isolates of clades 1–4, but weakly to clade 5; PspA2+4 and PspA2+5 sera had stronger binding to clade 5 isolates.
Chen et al., 2015 [34]	CD1 mice	PspA fragments: H70 (SM1 peptide+proline-rich region); CD2 (PRR only). PspA fused to the chimeric YLN protein (L460D+two CbpA domains: YPT and NEEK). PspA fused to L460D alone.	Protection against lethal challenge. Reduction in meningitis incidence Higher protection with H70+YLN formulation.
Converso et al., 2017 [35]	BALB/c mice	Chimeric protein rPspA-PotD+alum (s.c.).	Protection against lethal challenge with two pneumococcal strains (St A66.1 and ATCC6303); survival comparable to rPspA alone; rPotD alone not protective.
Yu et al., 2018 [36]	BALB/c mice	Bivalent vaccine: fusion protein PsaA-PspA23 (clades 2 & 3, family 1 & 2)+recombinant PspA4 (clade 4, family 2)+alum (s.c.).	Protection against lethal challenge with PspA clades 1–5; survival > 50% for all strains, up to 100% for some; significantly higher than PPV23 and PBS controls.
		Same bivalent vaccine (PsaA-PspA23+PspA4+alum).	Bacterial loads in blood reduced to <10 CFU/mL; near complete clearance for multiple clades; superior to PPV23.
Suzuki et al., 2021 [37]	BALB/c mice	Chimeric RBD-PspA protein (i.m.).	100% survival; high serum IgG levels against RBD and PspA. Class switching from IgM to IgG; Activation of PspA-specific helper T cells.
Chan et al., 2022 [38]	Mice and rabbits	Multiple-antigen vaccine (MAV) derived from *S. pneumoniae* TIGR4 lysates, enriched for surface proteins including PspA and pneumolysin	Induced broad antibody responses; passive transfer of immune rabbit serum protected against lethal sepsis caused by homologous and heterologous pneumococcal strains.
Afshari et al., 2023 [39]	BALB/c mice	Chimeric protein PspA1-5c+p fused with PhtD.	100% survival, increased protection when compared to individual antigens Increased bacterial clearance in the spleen. Reduction in bacterial load in blood.
Milani et al., 2023 [40]	BALB/c mice	Chimeric vaccine: N-terminal PspA fragments fused to non-toxic pneumolysin derivative (PlD)	Increased cross-protection against strains with heterologous PspAs; Enhanced complement C3 deposition on multiple strains; antibodies mediated complement activation; both PspA and Ply antigens contributed to protective effect.
Zane et al., 2023 [41]	BALB/c mice	rPspA–PdT; rPspA–RL–PdT; rPspA–FL–PdT Adjuvant: Al(OH)_3_, administered subcutaneously in three doses	Both FL and RL formulations conferred 100% protection against a lethal intranasal challenge with *S. pneumoniae* A66.1.
Dion and Ashurst, 2025 [42]	BALB/c mice	s.c. Choline binding proteins (CBPs) from WT and PspA negative *S. pneumoniae*	100% survival with CBPs including PspA. Efficacy varies according to the antigenic composition of the proteins extracted from each strain.
Vectored vaccines and nanoparticles			
Goulart et al., 2017 [43]	C57BL/6 mice	rBCG strains expressing PspA-PdT, SP0148, SP2108 (prime)+recombinant proteins (boost).	90% survival after lethal challenge (vs 100% mortality controls).
Castro et al., 2020 [44]	BALB/c mice	wPPspA4Pro (inactivated *B. pertussis* expressing Fha44:PspA4Pro)	Low anti-PspA4Pro IgG; no binding to native PspA; no complement deposition; no protection against lethal challenge.
		Purified PspA4Pro	High anti-PspA4Pro IgG; binding to native PspA; complement deposition; 60% survival after lethal challenge.
		Prime–boost protocol with wPPspA4Pro followed by PspA4Pro	Antibody levels similar to wPPspA4Pro; no improved binding or complement deposition; no protection.
		Purified Fha44:PspA4Pro	Similar IgG levels as PspA4Pro; weaker binding to native PspA; low complement deposition; no protection.
Figueiredo et al., 2022 [45]	BALB/c mice	PspA encapsulated or adsorbed in hybrid nanoparticles of PLGA/HCl-CS or PGA-co-PDL/HCl-CS	PLGA/HCl-CS/encapsulated PspA: 100% protection against lethal challenge; PGA-co-PDL/HCl-CS/adsorbed PspA: 83% protection; the encapsulated formulation promoted stronger dendritic cell activation and better preserved PspA structural integrity.
Majumder et al., 2024 [46]	Swiss Webster and C57BL/6 mice	Outer membrane vesicles (OMVs) from engineered *Y. pseudotuberculosis* expressing PspA (OMV-PspA, i.m.)	100% survival against secondary Spn D39 challenge (short-term); 80% long-term protection at day 205; serum transfer conferred 80% survival, confirming antibody-mediated protection.
			90% survival against secondary Spn A66.1 (serotype 3) challenge; cross-protection confirmed against multiple clinical isolates (high OPK activity).
Trentini et al., 2024 [47]	Neonatal C57BL/6 mice	rBCG PspA-PdT (prime) followed by rPspA-PdT+Al(OH)_3_ (boost).	100% survival; IgG1 to IgG2c switching; increased memory B/T cells; Enhanced pro-inflammatory cytokines (IL-6, IL-17, TNF-α, IFN-γ).
		rBCG PspA-PdT (prime only).	Partial protection; high-affinity antibodies; increased memory B/T cells; reduced total serum Ig.
		WT-BCG (prime) followed by rPspA-PdT+Al(OH)_3_ (boost).	Partial protection via BCG-trained immunity; Reduced memory B cells vs. rBCG PspA-PdT.
		rPspA-PdT+Al(OH)_3_ (two doses).	Low/non-significant protection; high IgG1 but poor bacterial binding.
Mucosal and combined immunization strategies			
Kang et al., 2002 [48]	Mice	Recombinant attenuated *Salmonella enterica* serovar Typhimurium (Δcya, Δcrp) expressing N-terminal PspA (oral).	Higher levels of systemic IgG and mucosal IgA; protection against pneumococcal sepsis
Campos et al., 2008 [49]	Mice	*Lactobacillus casei* expressing recombinant N-terminal PspA (clade 1) (intranasal)	Increased mucosal and systemic antibody responses; Enhanced C3 deposition on pneumococcal surface; increased protection against lethal challenge (A66.1); enhanced bacterial clearance by phagocytosis.
Li Y et al., 2009 [50]	Mice	Attenuated *Salmonella* with regulated delayed attenuation expressing PspA	Higher IgG levels; increased IL-4 and IFN-γ production; enhanced protection against lethal infection
Xin W et al., 2009 [51]	Mice	Recombinant *Salmonella* expressing hybrid PspA fragments (families 1+2).	Broad cross-protection against different pneumococcal families
Muralinath et al., 2011 [52]	Mice	*Salmonella*-derived outer membrane vesicles (OMVs) carrying PspA (intranasal)	Protection against lethal pneumococcal infection; safer alternative to live vectors
Frey et al., 2013 [28]	Healthy adults (Phase I clinical trial).	Recombinant attenuated *Salmonella typhi* expressing PspA (oral).	Safe and well-tolerated (up to 10^9^ CFU); no protective efficacy established; highlights need for optimization of antigen expression and immunogenicity.
Zhang et al., 2019 [53]	BALB/c mice	Recombinant PspA, delivered in a subcutaneous prime followed by an intranasal booster	The combined SC plus IN regimen provided 100% protection against lethal pneumococcal challenge, outperforming single-route immunization.
Tada et al., 2021 [54]	BALB/cCrSlc mice	Intranasal vaccine rPspA+pCA adjuvant	Increased survival in BALB/cCrSlc mice. Robust systemic and mucosal immune responses (IgG and IgA), predominant Th2 profile; combined formulation significantly superior to individual components.
Kono et al., 2023 [55]	BALB/cByJ mice	Recombinant PspA (PspA2/TIGR4) intranasally administered to adult females before mating, with cholera toxin subunit B adjuvant (first 2 weeks)	Offspring of immunized mothers: Increase in anti-PspA IgG-producing splenocytes; higher IgM, IgG1, IgG2a, IgG2b titers; increased IFN-γ, IL-17A; prolonged survival after lethal challenge.
		Recombinant PspA (PspA2/TIGR4) intranasally administered to adult females before mating, with cholera toxin subunit B adjuvant (first 2 weeks)	Offspring without maternal immunization or only breastfeeding: similar levels of anti-PspA IgG/cytokines; no improved survival.

s.c. =subcutaneous; i.m. = intramuscular; OPA = opsonophagocytosis.

**Table 2:** Studies evaluating the protective efficacy of PspA against pneumococcal pneumonia.

Study	Model	Formulation	Results
Roberts et al., 2019 [56]	BALB/c mice	rPspA or Prevnar (PCV7/PCV13), (i.m.)	Higher IgG levels in PspA 100% protection against A66.1 PspA significantly reduced bacterial load in BAL and lungs, with complete clearance in blood; increased protection against D39 with PspA
Kramskaya et al., 2019 [57]	Female BALB/c mice	Live attenuated influenza vaccine (LAIV), recombinant chimeric PSPF protein (PsaA, PspA, and Shr1875 associated with flagellin), i.n.	The combined LAIV+PSPF formulation significantly reduced pulmonary viral and bacterial loads. Higher IgG levels.
Wiedinger et al., 2020 [58]	C57BL/6 mice	PspA-IgG2a-Fc (adjuvant-free)	Effective protection; increased AM1; increased lung DC subsets; higher levels of Th1 cytokines; increased PspA-specific IgG/IgA.
		PspA-IgG1-Fc (adjuvant-free)	Minimal benefit vs. PspA alone; inhibitory FcγRIIB interaction.
	C57BL/6 mice (animal deficient for FcyRIIB)	PspA-IgG1-Fc in FcγRIIB KO	Enhanced protection; increased B cell maturation and proliferation.
Goulart et al., 2020 [59]	C57BL/6 mice	rBCG expressing PspA-PdT (prime dose) followed by rPspA-PdT+Al(OH)_3_ (boost dose)	Reduced bacterial load in BALF (~10^2^ CFU at 12h; clearance at 48h); no blood dissemination; increased neutrophil influx; increased CD4^+^ lymphocytes; higher anti-PspA IgG1/IgG2c; higher complement deposition.
Nakahashi-Ouchida et al., 2021 [25]	macaques	Trivalent PspA formulation in a cCHP nanogel delivered as nasal spray (5 doses in 2 weeks intervals)	Enhanced serum and lung IgG; enhanced mucosal IgA; higher complement deposition. Reduced lung inflammation and lower bacterial counts in the lungs (family 1 and 2 pneumococcal strains).
Dos Santos et al., 2022 [60]	BALB/c mice	Fusion protein rPspA-PlD1 (N-terminal PspA+pneumolysin derivative PlD1) with Al(OH)_3_ adjuvant	Reduced lung bacterial load (day 7, 1/7 animals are bacteria-free); increased IL-6, TNF-α; reduced leukocyte infiltration vs. control.
		PspA alone with Al(OH)_3_ adjuvant	Trend toward reduced bacterial load (not significant); no significant change in BALF cellular infiltrate.
		PlD1 alone with Al(OH)_3_ adjuvant	Similar lung bacterial loads vs. control; increased BALF leukocytes (6–24 h); no significant protection.
Yokota et al., 2023 [61]	Mouse C57BL/6	Fusion PspA+CpG and/or curdlan (prime i.m.+boost i.n.)	Higher Serum IgG and bronchoalveolar IgA; effective prevention of pneumococcal infection.
	Cynomolgus macaque	Fusion PspA+CpG and/or curdlan (prime i.m.+boost intratracheal)	Higher Serum IgG and bronchoalveolar IgA; effective prevention of pneumococcal pneumonia
Majumder et al., 2024 [46]	Swiss Webster and C57BL/6 mice	Outer membrane vesicles (OMVs) from engineered *Y. pseudotuberculosis* expressing PspA (OMV-PspA, i.m.)	100% protection (influenza–*S. pneumoniae* coinfection); reduced lung damage; reduced bacterial burden; balanced Th1/Th2; strong lung CD4+/CD8+ T-cell responses; increased opsonophagocytic activity
Ortiz Moyano al., 2024 [62]	Three-week-old female Swiss albino mice.	i.n. Pneumovax^®^ 23 (PPV23) or PSPF chimeric protein (PsaA–Spr1875–PspA–FliC), Combined with Cp 090104 or BLPs. 3 doses on days 0, 14, and 28;	Significant reduction in lung bacterial load in both primary and secondary pneumococcal infections. PSPF+Cp 090104 produced the strongest mucosal immunity (IgA/IgG) and the most effective pneumonia protection.
Rodrigues et al., 2024 [63]	BALB/c mice	Formulation: NCMPs carrying liposomes with PspA1 or PspA4Pro (6 μg total protein) Dosing schedule: Two doses, administered 15 days apart. Intranasal	Female BALB/c mice intranasally immunized with NCMP–liposome formulations containing PspA1 or PspA4Pro (two doses, 15 days apart) exhibited cross-protection against pneumococcal pneumonia, with reduced lung bacterial load, mucosal IgA/IgG induction, and formation of CD4^+^ TRM cells

i.m. = intramuscular; i.n. = intranasal; s.c. = subcutaneous.

**Table 3:** Studies investigating PspA as a vaccine against otitis media.

Study	Model	Formulation	Results
White et al., 1999 [64]	Sprague-Dawley rats	Infection with *S. pneumoniae* serotype 6A into the middle ear cavity after surgical exposure of the tympanic bulla Immunization with rPspA in Freund’s adjuvant (s.c.)	None of the immunized animals developed otitis, whereas control groups showed a high incidence; protection was associated with high levels of specific IgG, demonstrating that the humoral response induced by PspA was sufficient to prevent middle ear infection.
Schachern et al., 2008 [65]	Chinchillas	Intrabullar inoculation with *S. pneumoniae* D39 Wild-type *S. pneumoniae* D39 (serotype 2) and isogenic mutants (ΔPspA, ΔpspC, ΔPspA/ΔpspC)	Increased bacterial loads and inflammation in wild-type and PspC^−^ groups; undetectable bacteria and reduced inflammatory responses in PspA-deficient groups. Findings demonstrate the critical role of PspA in pneumococcal otitis media.
Tsuprun et al., 2008 [66]	Chinchilla	Inoculation of middle ear with WT *S. pneumoniae* D39 or isogenic mutants (ΔPspA, Δply)	WT caused permanent hearing loss (10–15 dB at 4–32 kHz, >20 dB at 1–2 kHz) and inner ear pathology. ΔPspA or Δply mutants caused no significant hearing loss.
Li-Korotky et al., 2010 [67]	Human middle ear epithelial cell line (HMEEC)	Expression analysis of NanA, HylA, PspA, CbpA under simulated middle ear conditions: normal, Eustachian tube obstruction (ETO), and tympanostomy tube (TT) placement	Transparent (T) phenotype: higher PspA expression; opaque (O) phenotype: higher CbpA. Pathological conditions (ETO, TT) enhanced NanA, HylA, and PspA expression. Suggests PspA is a signature virulence factor of T variants during OM pathogenesis.
Schachern et al., 2013 [68]	Chinchilla	WT *S. pneumoniae* D39 (serotype 2) and isogenic mutants (ΔPspA, Δply, ΔPspA/Δply)	ΔPspA: no viable bacteria detected in middle ear at 48 h, rapid clearance, least virulent. Δply: CFUs remained near inoculum level, attenuated pathology. ΔPspA/Δply: similar virulence to WT, no attenuation. WT: highest CFU and inflammation.
Schachern et al., 2014 [24]	Chinchilla	Middle ear inoculation with *Streptococcus pneumoniae* D39 WT and isogenic mutants (ΔPspA, ΔpspC, ΔPspA/ΔpspC); no vaccination was performed	ΔPspA and ΔPspA/ΔpspC mutants showed no bacterial growth, strongly attenuated infection, and minimal inflammation; the ΔpspC mutant displayed reduced but still detectable bacterial load; the wild-type strain caused the highest levels of inflammation and penetration into the round window membrane and inner ear structures
Habets et al., 2016 [69]	BALB/c and C57BL/6 mice (WT, IL-17RA^−/−^, and antibody-deficient μMT	Intranasal recombinant PspA (from TIGR4)+cholera toxin subunit B (CTB)	Significant reduction in pneumococcal load in middle ear and nasal cavity (>1 log). Protection required IL-17 signaling but not antibodies; PspA alone induced antibodies but no protection.

s.c. = subcutaneous; WT = wild type.

**Table 4:** PspA as a vaccine candidate against pneumococcal colonization.

Study	Model	Formulation	Results
Fukuyama et al., 2015 [70]	macaques	cCHP-PspA nanogel delivered in an intransal spray; five doses in two weeks intervals	Enhanced IgG and mucosal secretory IgA; passive protection; passive protection of mice against intravenous pneumococcal challenge; Th2 and Th17 cytokine responses and increased levels of miR-181a and miR-326 in serum and respiratory tract.
Tostes et al., 2017 [71]	C57BL/6 mice	Intranasal rPspA1, rPspA2, rPspA3, rPspA4 (isolated or combined)+whole-cell pertussis vaccine (wP)	Significant reduction in colonization; best results with rPspA1 or rPspA4+wP; mixture rPspA1+rPspA4+wP showed stronger reduction than single antigens; no strain replacement observed.
Kuipers et al., 2017 [72]	C57BL/6J, BALB/c, and CB6F1 (7-week-old female mice)	rPspA (TIGR4)—3 doses at 2-week intervals	Significant reduction in nasal colonization across all strains; strongest protection in CB6F1, where nearly all vaccinated animals showed undetectable colonization.
Kuipers et al., 2017 [73]	Mice (primarily C57BL/6)	Intranasal immunization with OMVs from attenuated *Salmonella typhimurium* (SL3261ΔtolRAΔmsbB) displaying the PspA α1–α2 fragment; control animals received OMVs lacking antigen	Significant protection against colonization by TIGR4 and high IL-17A strains; no protection against low IL-17A strains. Protection correlated with elevated IL-17A levels in the nasal mucosa.
Converso et al., 2017 [35]	BALB/c mice	Chimeric protein rPspA-PotD+alum (s.c.)	Significant reduction in nasopharyngeal colonization (~1 log); effect not seen with rPspA alone; rPotD also reduced colonization.
Goulart et al., 2017 [43]	C57BL/6 mice	Same rBCG mix+protein boost	Significant reduction in colonization load vs. controls.
Colichio et al., 2020 [74]	C57BL/6 mice	Intranasal rPspA1+rPspA4+whole-cell pertussis vaccine (wP); compared with PCV13 (s.c.) and combination (PspA1+4+wP+PCV13)	PCV13: reduced VT strain, but gave competitive advantage to NVT. PspA1+4+wP: reduced both VT and NVT strains. Combination (PspA1+4+wP+PCV13): reduced both strains, overcoming serotype replacement.
Kaplonek et al., 2022 [75]	Female BALB/c mice	PS14-mPspA4Pro conjugate (PspA4Pro+capsular polysaccharide serotype 14) or co-administered unconjugated antigens; (s.c.)	Reduction in nasal colonization. Cross-protection with PspA4Pro against colonization by a heterologous strain.
Carneiro et al., 2025 [76]	BALB/c mice	pEVs (WT or ΔPspA) (i.n.)	Both WT and ΔPspA pEVs reduced nasal colonization (~1 log) vs. controls; PspA not essential for colonization protection.

s.c. = subcutaneous; i.n. = intranasal; WT = wild type.

## Data Availability

No new data were created or analyzed in this study. Data sharing is not applicable to this article.
